# Contrasted *NCED* gene expression across conifers with Rising and Peaking abscisic acid responses to drought

**DOI:** 10.1016/j.stress.2024.100574

**Published:** 2024-12-01

**Authors:** Gabriele Rizzuto, Dapeng Wang, Jinhui Chen, Tin Hang Hung, Anne Charlott Fitzky, Emily Flashman, John J. MacKay

**Affiliations:** 1 Department of Biology, University of Oxford, South Parks Road, Oxford OX1 3RB, UK; 2 National Heart and Lung Institute, Imperial College London, London, SW3 6LY, UK; 3 Sanya Nanfan Research Institute of Hainan University, Hainan Yazhou Bay Seed Laboratory, Sanya, 572019, People’s Republic of China; 4 Department of Forest and Soil Sciences, Institute of Forest Ecology, University of Natural Resources and Life Sciences (BOKU), Peter-Jordan-Straße 82, A-1190 Vienna, Austria

**Keywords:** water stress, abscisic acid metabolism, RNA transcript level, gene regulation, enzyme sequence evolution

## Abstract

Conifer trees have diverse strategies to cope with drought. They accumulate the plant hormone abscisic acid (ABA) following a range of profiles from constantly rising to peaking and falling (R- and P-type) with direct effect on foliar transpiration. The molecular basis of this adaptive diversification among species is largely unknown. Here, we analysed the sequences of candidate ABA biosynthesis and catabolism genes and monitored their expression in response to intensifying drought. We studied young trees from Cupressaceae, Pinaceae, and Taxaceae under controlled drought conditions and compared changes in water status, ABA profiles and gene-specific transcript levels. Our data indicate that R-type and P-type ABA profiles may be controlled by divergent expression of genes involved in the biosynthetic and catabolic pathways of ABA, respectively, and emphasize a key role of nine-*cis*-epoxycarotenoid dioxygenases (*NCED*) genes. Our results open the doors to understanding the molecular basis of contrasted drought response strategies across conifer taxa, which we expect will help foresters grow more drought-resilient trees.

## Introduction

Climate change represents a threat to forest ecosystems worldwide (Masson-Delmotte *et al*., 2019) with phenomena such as drought-related forest die-off becoming more widespread (Allen *et al*., 2010; McDowell *et al*., 2016). The increasing frequency, duration, and intensity of droughts has the potential to alter forest structure, functioning, and composition in coming decades (Williams *et al*., 2012; Allen, Breshears and McDowell, 2015; McDowell *et al*., 2016). The functional specializations and differential survival to extreme events among species may ultimately drive tree mortality and related changes in forest characteristics (O’Brien *et al*., 2017; Anderegg *et al*., 2016; Russell *et al*., 2014). Plant species possess diverse and contrasting water use strategies to cope with drought (for a review, see Volaire 2018), which can be useful to explain or even predict the relative vulnerability to drought of different tree species (Blackman *et al*. 2019; Skelton *et al*,. 2015). Trees have adapted to drought conditions with mechanisms to manage water and carbon resources, mainly by avoiding catastrophic failure of their transportation and exhaustion of their pools within plant tissues (McDowell *et al*., 2022). Some mechanisms such as the regulation of transpiration are well-studied in trees (Blackman *et al*. 2019; Skelton *et al*,. 2015) but the molecular basis of diversification among species is largely unknown, with most of the research on genes and metabolic regulation more focused on model and crop plants.

The key hormone coordinating plant responses to drought is abscisic acid (ABA), which controls osmotic relations in several processes, such as growth, dormancy, and stress responses. A detailed understanding of the role of ABA in plant responses to water deficit has developed from studies in model plant systems (Seo and Marion-Poll, 2019; Hsu *et al*., 2021). The concentration of ABA increases during water stress (either soil water or air humidity) (Tardieu and Davies, 1993; Sussmilch, Brodribb and Scott A. M. McAdam, 2017), triggering complex signalling that induces guard cells to shrink and the stomatal pores to close (Kollist, Nuhkat and Roelfsema, 2014; Hsu *et al*., 2021). Members of the 9-cis-epoxycarotenoid dioxygenases (NCED) protein family have emerged as a key regulatory step in the ABA biosynthetic pathway. NCEDs catalyse the first committed step of ABA biosynthesis, i.e., cleavage of 9-cis-violaxanthin or 9-cis-neoxanthin to produce xanthoxin (Burbidge *et al*., 1997; Schwartz *et al*., 1997; Tan *et al*., 1997; Qin and Zeevaart, 1999; Iuchi *et al*., 2001), and their expression is up-regulated within minutes from the imposition of drought stress in several angiosperms (Burbidge *et al*., 1997; Tan, Cline and McCarty, 2001;

Hanada *et al*., 2011; Hauser, Waadt and Schroeder, 2011). ABA biosynthesis includes other drought responsive enzymes. The first enzyme in the pathway is the zeaxanthin epoxidase (ZEP) (AtABA1 in *A. thaliana*), which is responsible for the epoxidation of zeaxanthin to all-trans-violaxanthin, then converted to neoxanthin and violaxanthin, the substrates of NCEDs (Marin *et al*., 1996; Audran *et al*., 1998, 2001). The very last enzyme in the pathway is also drought responsive and catalyses the oxidation of abscisic aldehyde to ABA (encoded by *AtAAO*3 *A. thaliana*) (Seo *et al*., 2000a; Koiwai *et al*., 2004). See Ma *et al*. (2018) for a thorough review of the ABA biosynthesis pathway.

Conifers form an understudied group of plant species (with 600+ taxa) which are of interest to understanding the evolution and diversification of mechanisms to cope with drought, including the role of ABA. In contrast to angiosperms, conifers may upregulate their foliar ABA levels only in response to prolonged water deficits (McAdam and Brodribb, 2015). During short-term or mild drought episodes such as daily fluctuations in air humidity, stomatal closure is driven by passive changes in the leaf water status (Brodribb and McAdam, 2013; McAdam and Brodribb, 2016). Conifers appear to be able to switch between these two mechanisms of stomatal control, and this ability may have been under natural selection. Basal conifer clades, such as Pinaceae, possess low cavitation-resistant xylem and respond to prolonged drought stress by accumulating high levels of foliar ABA and tightly closing their stomatal pores (ABA Rising-type response). In contrast, the xylem of the more derived clades in the Cupressaceae and Taxaceae is resistant to cavitation, and some species switch from ABA-driven to water potential-dependent stomatal closure (ABA Peaking-type response), allowing some degree of gas exchange until the advanced stages of drought (Brodribb *et al*., 2014; Harfouche, Meilan and Altman, 2014).

The recent advances in the understanding of drought responses across conifers open the possibility to study the genes involved in ABA regulation. Although ABA accumulation can be released from conjugated stores of ABA-GE, here we focus on genes that may support *de novo* biosynthesis of ABA, such as *NCEDs*, and catabolism, mainly ABA 8’-hydroxylases encoded by cytochrome P450 (*CYP707A*) genes. Transcriptome profiling studies and targeted gene expression analyses have indicated that ABA metabolism is conserved in conifers and identified the genes that are responsive to drought stress (Haas *et al*. 2021, Pashkovskiy *et al*. 2019, Du *et al*. 2018, Fox *et al*. 2017); however, they have mainly analysed Pinus and Picea species (Pinaceae), which typically have a R-type response. An ABA signalling gene was shown to be functionally conserved between conifers and flowering plants (Zeng *et al* 2012). We hypothesise that one or more *NCED* genes may be responsible for regulating ABA profiles in conifers. We predict that changes in transcript levels of genes encoding NCEDs are key to Peaking and Rising phenotypes (P-type and R-type). This hypothesis assumes that ABA catabolism is constitutive or enhanced during drought, as already reported for other plants (Kushiro *et al*., 2004). By improving our understanding of the mechanisms that underpin these varied responses, we may develop new insights with practical implications for species choice and silviculture, for molecular tree breeding including genomic selection, and for bioengineering approaches.

## Materials and methods

### Transcriptomic sequencing and assembly

We used a preliminary drought experiment - run similarly to those reported below - to develop *de novo* transcriptome assemblies, support gene discovery work in diverse species and identify reference genes for targeted gene expression analyses, described here (for details see Supplementary materials – I. Previous drought experiment and transcriptome work).

### Gene sequence analyses

Sequence searches were conducted to analyse several genes in the ABA biosynthetic and catabolic pathways including orthologues sequences of model *Arabidopsis thaliana ABA1, AAO3, CYP707A3, NCED3*. We used three new conifer transcriptomes *Chamaecyparis lawsoniana, icea. sitchensis* and *Taxus baccata* (described here) along with those of *Larix laricina, Picea glauca, Thuja occidentalis* (van Ghelder *et al*., 2019), and of the ancient gymnosperm *Ginkgo biloba* available at NCBI. Protein sequences were also included from other plant taxa as follows: *Amborella trichopoda, Arabidopsis thaliana, Marchantia polymorpha, Oryza sativa, Physcomitrella patens* and *Selaginella moellendorffii*. RNA sequences were translated to amino acid sequences using VirtualRibosome – 2.0 (DTU Health Tech) and clustered on CD-HIT, using 97% identity threshold to filter highly similar sequences out. Sequences shorter than 200 amino acids were removed to improve phylogenetic outputs. Putative ortholog sequence detection was then conducted with Orthofinder – 2.3.1 (Emms and Kelly, 2019). *A. thaliana* TAIR accessions for selected candidate genes from the ABA biosynthetic and catabolic pathways (*ABA1, AAO3, CYP707A3* and *NCED3*) were used as references for conifer ortholog identification within orthogroups (see Supplementary table 3 for gene IDs and further details). Orthogroup sequences clustered by the Orthofinder algorithm were used for further phylogenetic analyses through MEGA X (Molecular Evolutionary Genetics Analysis, (Kumar *et al*., 2018)). First, a new MSA (Multiple Sequence Alignment) was generated in MAFFT (Multiple Alignment using Fast Fourier Transform, (Katoh *et al*., 2002)) with 1000 iterations. This MSA was used for phylogenetic tree inference in MEGA. A Maximum Likelihood tree was inferred using JTT (Jones, Taylor and Thornton, 1992) substitution model and node support values were obtained by using 1000 bootstrap replicates.

### Plant material and experimental design

Three different drought trials were run between March and October 2019 including eight different conifer species and one hybrid (see Table 1, for details) by using one- to two-year-old conifer seedlings 15-40 cm tall, from commercial tree nurseries (see Supplementary table 4 for species identity, provenance, and provider). The species included *Chamaecyparis lawsoniana, Juniper communis, Picea sitchensis, Picea sitchensis × Picea glauca, Taxus baccata, Larix eurolepis, Pseudotsuga menziesii, Tsuga heterophylla, Sequoia sempervirens*. Trees were transferred to 3 L pots containing a 3:1 volumetric mix of potting compost (M2, ICL, Everris ltd) and horticultural grit sand (Bathgate horticulture) and were acclimated for 4-6 weeks in a greenhouse with natural light supplemented to achieve a photoperiod of 16h/8h day/night and with temperature set to 24/16 °C (day/night). Cooling and humidity control was achieved through venting via roof windows.

Each experimental trial used a complete randomised split-plot block design, with the split-plots containing either the drought stress treatment and well-watered controls. The drought treatment consisted of completely withholding watering. Trial 2 was shorter due to higher summer temperatures, which exceeded the cooling capacity of the greenhouse.

### Sampling and tree physiology measurements

Samples were collected for measurements between 11 am and 2 pm from randomly selected plants across all plots and blocks; the number of biological replicates (one sample per tree) was either four (trials 1 and 2) or six (trial 3) for both the well-watered and drought-stressed trees at each time point. The volumetric Soil Water Content (SWC) was recorded in individual pots during sampling by completely inserting a soil moisture probe (ML3 ThetaProbe Soil Moisture Sensor, Delta-T Devices) into the potting mix at root depth and measurements were stored in a hand-held device (HH2 Soil Moisture Meter, Delta-T Devices). Twigs were excised by using a razor blade and stored in moist sealable plastic bags kept in a cold cooler until water potential measurements. Shoot tips with new fully expanded foliage were collected for molecular analyses; they were snap-frozen in 15 mL falcon tubes in liquid N and stored in -80 °C until analysis. Midday Shoot Water Potential (MWP) was measured by using a Scholander-type digital pressure chamber (SKPM 1400/80, Skye instruments). Pressure readings (bar) were recorded at the first appearance of xylem sap from the cut surface with the help of a hand-held lens. When tissues appeared dry and sap could not be clearly observed at the cut surface, measurements were considered inaccurate, and samples retained only for gene expression analysis.

### ABA extraction and quantification

ABA was extracted according to (McAdam, 2015) from four (trial 1 and 2) or six (trial 3) individual biological replicates. All procedures were performed under dim light to minimise ABA isomerisation.

HPLC analysis was carried out on a Waters Acquity H-class separation module. Separation was achieved on an ACE C18, 3 μm, 150 x 3 column (Hichrom, UK) maintained at 45 °C. Samples were eluted in A: 0.1 % acetic acid in H2O, B: 100% acetonitrile with a flow rate of 0.25 mL/min, a linear gradient of 20-95 % B in 5 min, and a total run time 8 min. Analytes were measured by mass spectrometry with a Waters Acquity TQD detector with electrospray ionisation in negative ionisation mode. ABA was detected at multiple reaction monitoring (MRM) of 262.8 > 152.7 and d6-ABA was detected at MRM of 268.8 > 158.7, retention time 3.9 min for both. A calibration curve was run in the system and then spiked with the same concentration of internal standard (d6-ABA). The calibration curve was then plotted as peak area for ABA/peak area for IS against ABA concentration.

### RNA isolation, primer design and RT-qPCR

Total RNA was isolated from 30-90 mg of frozen leaf tissue samples from *C. lawsoniana, P. sitchensis* and *T. baccata* collected in trial 1 (four individual biological replicates each), based on the observation of ABA P-type and R-type profiles during experimental procedures and to encompass a range of taxa. The tissue was ground to a powder in 2 mL tubes containing stainless steel beads and using a mixer mill without allowing them to defrost. RNA extraction and purification steps were performed according to the Monarch Total RNA Miniprep kit (NEB #T2010) by using the ground tissue either directly or after a pre-wash with a sorbitol solution for difficult samples yielding low quality RNA (see below) according to (Inglis *et al*., 2018). RNA was quantified both with a NanoDrop One Microvolume UV-Vis Spectrophotometer (Thermo Fisher) and a Qubit 4 Fluorometer (Thermo Fisher). RNA quality was determined from UV absorbance ratios 260/280 ~ 1.8-2 and 260/230 ~ 1.7-2. RNA integrity was assessed using a Bioanalyzer system (Agilent); samples with a RIN (RNA Integrity Number) ~ 7 or above were used in downstream analyses. Complementary DNA (cDNA) was synthesised from ~160 ng of total RNA using a Reverse Transcription Master Mix provided by Fluidigm according to manufacturer’s instructions.

Primers were selected using PrimerIdent online tool (Pessoa, Pereira and Teixeira, 2010) for multi-gene families or PrimerBlast (see Supplementary table 3 for primer details). Selected primers were tested *in silico* for tertiary and quaternary structures with OligoEvaluator from Sigma-Aldrich. Primers were obtained from Sigma and resuspended in TE buffer (Tris 10 mM, EDTA 1 mM) pH 8.0 (Teknova, #T0225) and tested by PCR amplification for specificity, visualised by gel electrophoresis and confirmed by Sanger sequencing. Newly synthesised cDNA was purified, diluted, and used as template in a pre-amplification reaction according to the Delta Gene Assays protocol (Fluidigm, South San Francisco, California, USA). Master mixes were prepared containing species-specific primers for the three test species (*C. lawsoniana, P. sitchensis* and *T. baccata*). Reverse-transcription quantitative PCRs (RT-qPCRs) were set up by using microfluidic 192.24 IFC (Integrated Fluid Circuits) plates on Juno system (Fluidigm) and then run using a Biomark HD system (Fluidigm) equipped with a high sensitivity laser imaging system. Rt-qPCR cycle threshold (Ct) values were normalised against well-watered control samples and housekeeping genes and used to calculate gene expression fold-change across time points. These values were then log2-transformed.

### Reference genes for RT-qPCR analysis

Candidate reference genes were identified from previous RNA-seq trials in *C. lawsoniana, P. sitchensis* and *T. baccata* including several tissues and drought treatments similar to those used here (see above). The FPKM (Fragments Per Kilobase Million) data were analysed with a R script pipeline, with three criteria for the identification of suitable reference genes: 1. Expression in all tissues and treatments; 2. Low variance in expression levels (log2(fold-change) between 0 and 1); 3. Medium to high expression levels (log2(mean(FPKM)) between 5 and 7).

Sequences with an FPKM < 1 were not included in the analysis as they were considered too low to accurately predict gene expression levels. This sequence search produced a total of three candidate housekeeping genes shared by our study species according to TAIR annotations: mRNA capping enzyme family protein (Lee *et al*., 2012), LisH dimerisation motif (Liu and Meyerowitz, 1995), RING/U-box superfamily protein (Kosarev, Mayer and Hardtke, 2002). The first two were used in downstream applications, as RING/U-box superfamily protein did not amplify reliably in PCR assays.

### Statistics and data analysis

All data analyses were performed in R environment (version 4.3.2, R Core Team), using RStudio RStudio Team (2020). SWC and MWP readings were tested for normality and homogeneity of variance considering treated and control trees as separate groups. Differences in mean SWC, mean MWP and ABA between well-watered control and drought-treated pots were assessed with a Student’s T test (*P < 0.05*).

Second order polynomial equations were fit to model the different relationships between shoot MWP and pot SWC. The slope “σ” of the linear regression between MWP and SWC was instead chosen to represent the stringency of water conservation strategy, in a way similar to (Martinez-Vilalta *et al*., 2014). In orther to display the varying degrees of drought sensitivity we plotted the linear regression between intercept and slope estimates of the linear model fitted to SWC and MWP.

Relations between ABA and MWP were analysed by fitting a smoothing curve to data and calculating its 95% confidence interval. When ABA records reached approximately the same levels as the starting record, the trend was a P-type, otherwise R-type.

A one-sample T test was implemented to compare the gene transcript levels detected for each target gene to a zero-fold-change baseline at each time point, equal to no change in gene expression (all significance levels are set to P < 0.05).

## Results

### ABA related gene sequence analysis

Sequence searches were conducted to analyse conifer genes in ABA biosynthesis and catabolism, including *aba1, aao3, cyp707a3, nced3* (See methods for details). We mined new reference transcriptomes that we developed in *C. lawsoniana, P. sitchensis* and *T. baccata* (for details see Supplementary materials – I. Previous drought experiment and transcriptome work) and public data-bases for both conifers and angiosperms.

**Figure 1:**
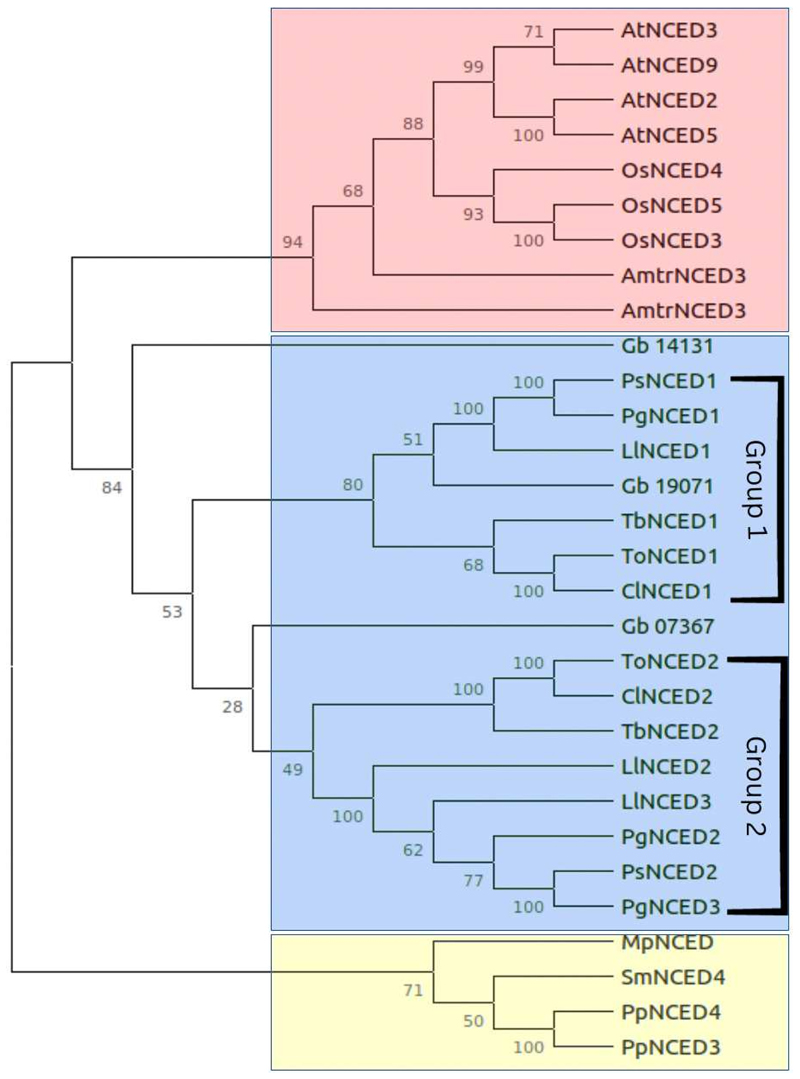
Putative **NCED** orthologs ML phylogenetic tree. Support bootstrap values (1000 replicates) are shown on the nodes. **Red**: angiosperms; **Blue**: gymnosperms; **Yellow**: seedless plants. **Amtr**: A. trichopoda; **At**: A. thaliana; **Cl**: C. lawsoniana; **Gb**: G. biloba; **Ll**: L. laricina; **Mp**: M. polymorpha; **Os**: O. sativa; **Pg**: P. glauca; **Ps**: P. sitchensis; **Sm**: S. moellendorffii; **To**: T. occidentalis.

We retrieved two putative conifer NCED protein sequences for each *C. lawsoniana, P. sitchensis, T. baccata* and *T. occidentalis*, and three sequences for *L. laricina* and *P. glauca*, which clustered into two distinct groups of conifer specific sequences – with a 53% bootstrap support value at the branch node (Figure 1) - here named group 1 and 2 (conifer sequences NCED1 to NCED3). A multiple sequence alignment (MSA) showed that NCED protein sequences were highly conserved from residue 98 to 609, with a global similarity score above 0.7 spanning the main catalytic domain (Supplementary figure 1).

We found one putative ABA1 sequence based on homology to the *A. thaliana* gene in *C. lawsoniana, P. sitchensis, T. baccata* and *T. occidentalis*, and two homologous sequences in *L. laricina* and *P. glauca* (Supplementary figure 2). The sequences were highly conserved (> 0.7 similarity global score) from residue 91 to 659, which includes characteristic FAD (flavin-adenine dinucleotide) binding domain and the FHA domain (forkhead-associated domain) (Supplementary figure 3). We also found one putative homolog sequence of AtAAO3 for each of the three study species, two for *L. laricina* and *T. occidentalis* (Supplementary figure 4). The conservation among these sequences was lower, especially within the aldehyde oxidase and xanthine dehydrogenase, a/b hammerhead domain (Ald_Xan_dh_C, residues 579 to 688). There was a higher degree of variation within the Molybdopterin cofactor-binding domain (residues 977 to 1239). The only recovered *P. sitchensis* sequence *(*PsAAO) was truncated, lacking the residues preceding the Ald_Xan_dh_C (Supplementary figure 5).

Our search for genes encoding enzymes involved in ABA catabolism focused on *CYP707A3* sequences, shown to control the main catabolic pathway of ABA in flowering plants (Kushiro *et al*., 2004). We found four putative CYP707A protein sequences for *C. lawsoniana*, three for *L. laricina*, two for *P. sitchensis* and only one for *P. glauca, T. occidentalis* and *T. baccata* (Supplementary Figure 6). The phylogenetic reconstruction indicated that the sequences formed two separate groups: group 1 containing ClCYP707A1, PsCYP707A1 and TbCYP707A1 and group 2 containing ClCYP707A2, ClCYP707A3 and ClCYP707A4. ClCYP707A2 and ClCYP707A3 further diverged from ClCYP707A4. Group 1 was more similar to AtCYP707A1 and AtCYP707A3, while group 2 was more similar to AtCYP707A2. Overall, we observed a high degree of sequence similarity among taxa (Supplementary Figure 7).

### Species differences in shoot water potential in response to soil desiccation

**Figure 2:**
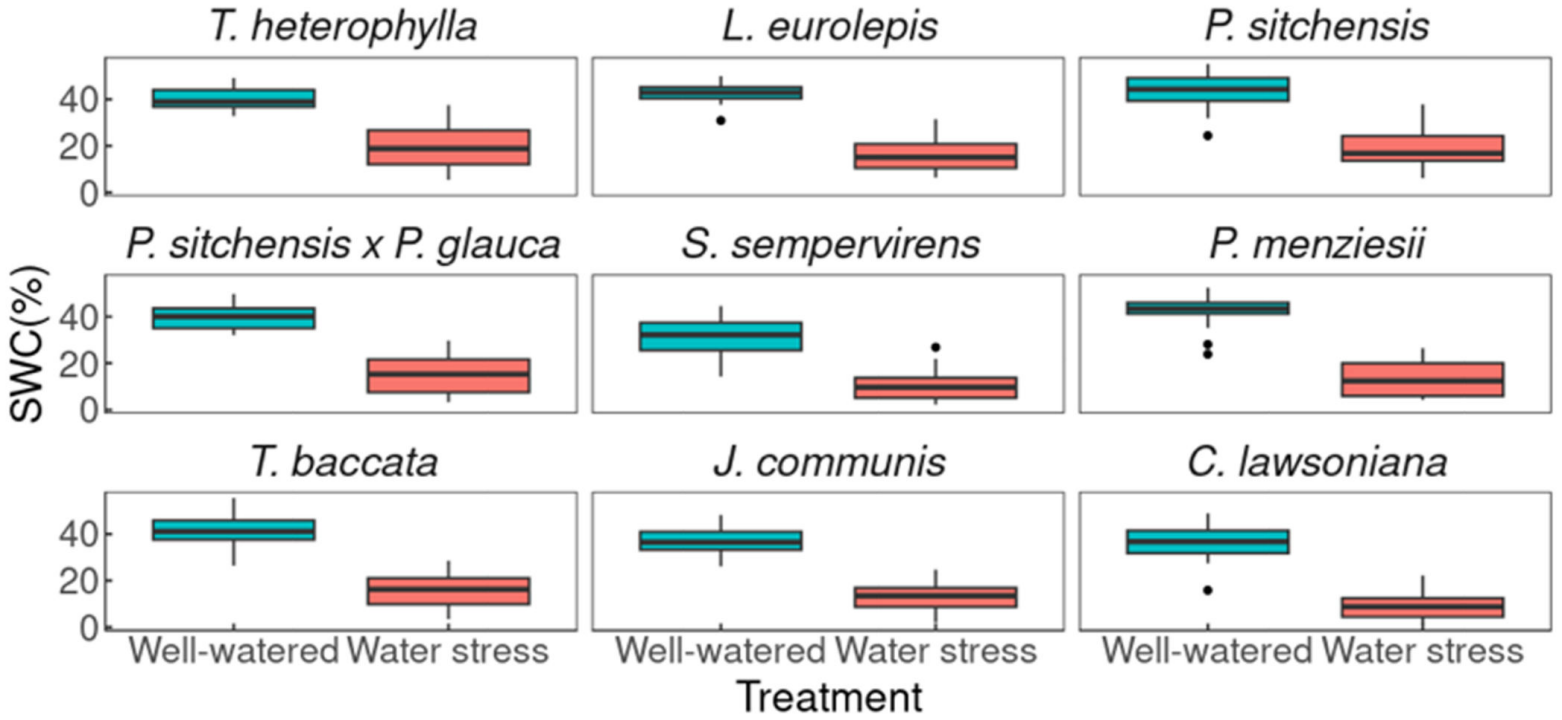
Differences in mean **SWC** in pots of droughted and well-watered plants. **Blue**: drought-treated trees, N = 16-32, depending on experimental design; **Red**: well-watered control trees, N = 16-32, depending on experimental design. **Asterisks:** significant differences between treated and control trees; **dots** represent potential outliers; **Boxes** indicate median, 25% and 75% quantiles **Bars** indicate maximum and minimum values. Student’s t-test (P value < 0.05).

**Figure 3:**
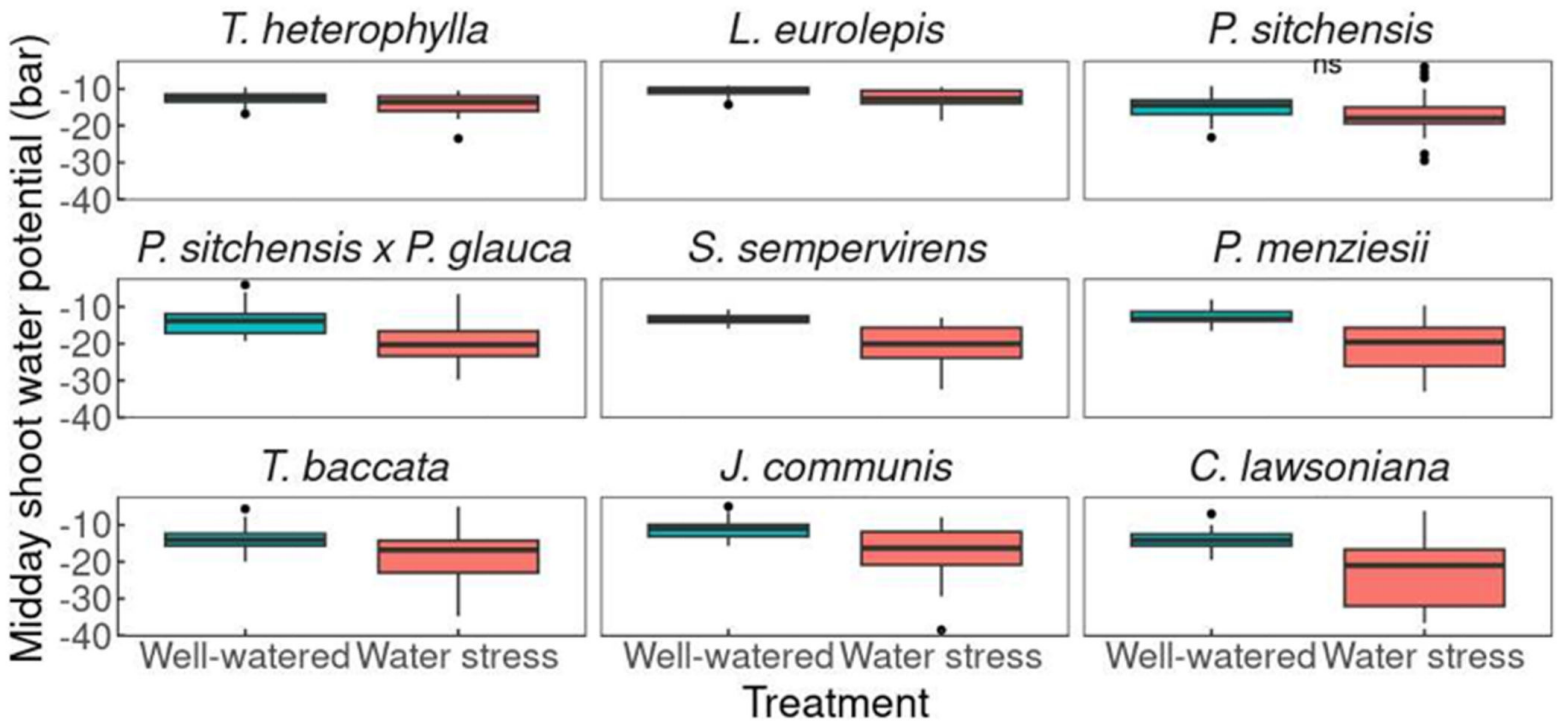
Differences in mean **MWP** between droughted and well-watered plants. **Blue**: drought-treated trees, N = 16-32, depending on experimental design; **Red**: well-watered control trees, N = 16-32, depending on experimental design. **Asterisks:** significant differences between treated and control trees; **dots** represent potential outliers; **Boxes** indicate median, 25% and 75% quantiles **Bars** indicate maximum and minimum values. Student’s t-test (P value < 0.05).

Controlled drought treatments were used to investigate the relationship between water relations, ABA accumulation, and gene expression in nine different conifer species. All droughted plants had a major decrease in SWC compared to the control plants (P < 0.05, see Figure 2). Water relations were monitored by following changes in the shoot MWP, which decreased with drought progression but to variable levels across species (Figure 3). The data show that *J. communis* reached as low as – 38.5 bar at 33 days of drought stress, and *P. sitchensis* had a highly variable response.

**Figure 4:**
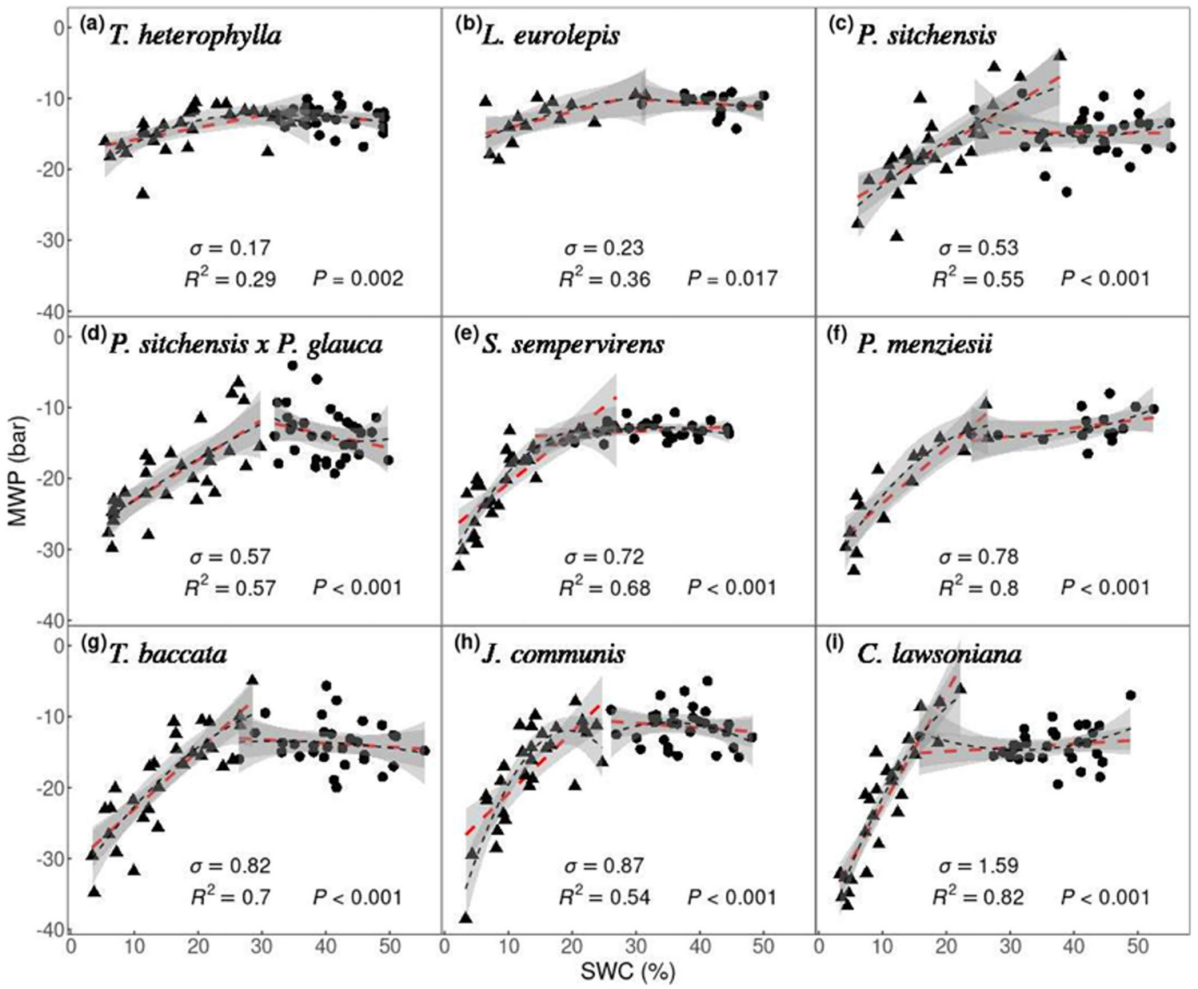
Curvilinear and linear relationships between MWP and SWC. **Circles**: well-watered controls, N = 16-32; **triangles**: drought-treated trees, N = 16-32. **Red line**: Linear regression model of MWP against % SWC. **Black dashed line**: second order polynomial regression. R^2^, P-values and slope for the ordinary linear model for drought-treated trees are shown. **Grey** areas are 95% confidence intervals.

We fitted an ordinary least square (OLS) regression to predict MWP with Species and SWC (formula: MWP ~ Species * SWC). The modelled relationship was statistically significant and explained a substantial proportion of the variance (Figure 4), with statistically significant effects of Species on MWP and of the interaction between SWC and Species, except in *T. heterophylla* and *L. eurolepis* (Supplementary table 5). We used the slope “σ” of the linear model estimated by OLS to quantify species-specific responses to drought stress. Its value ranged from σ = 0.17 in *T. heterophylla* to σ = 1.59 in *C. lawsoniana* (Figure 4).

**Figure 5:**
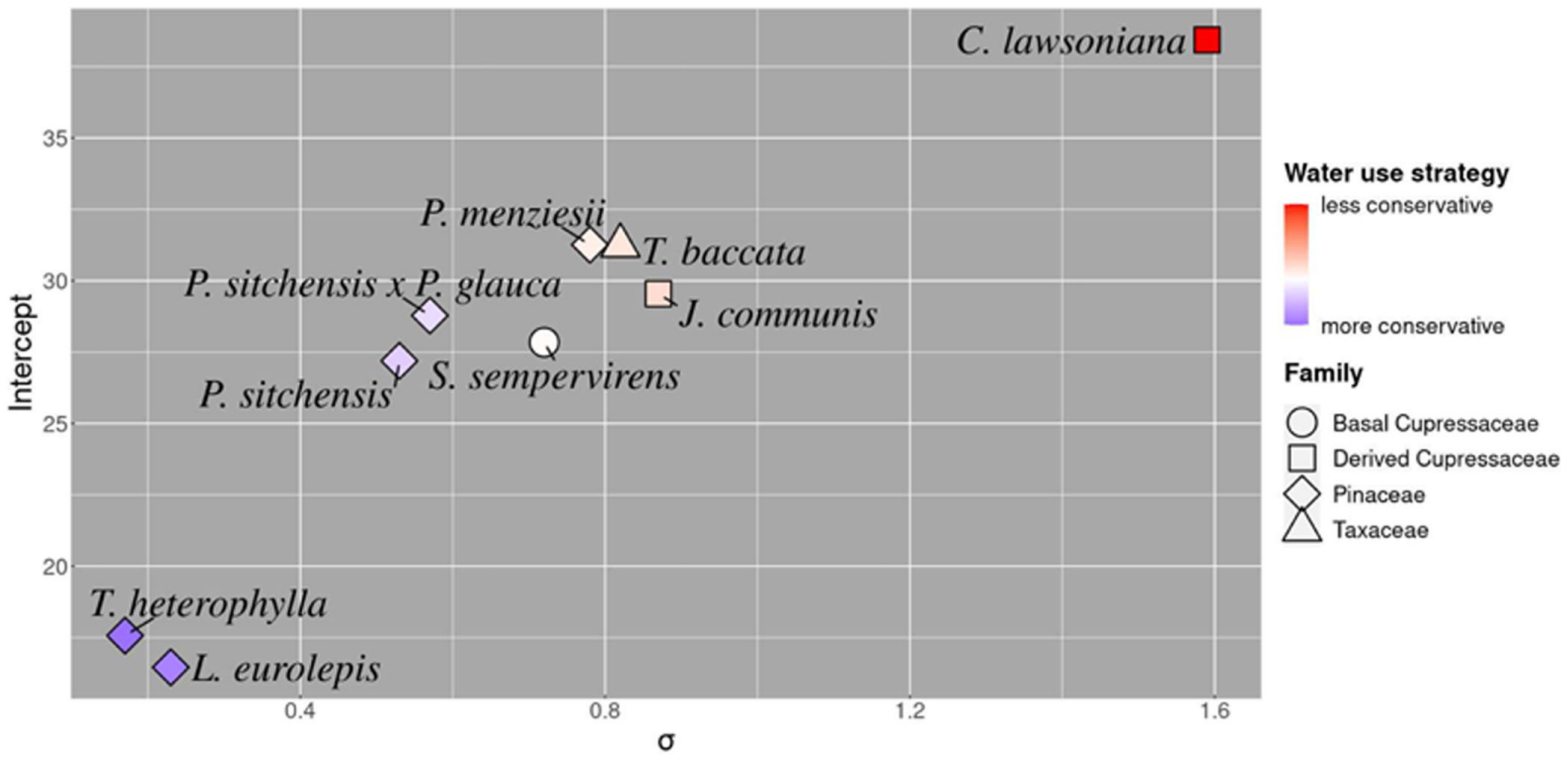
Two-dimensional spectrum of water use responses across conifer families. Intercept values (**intercept**) were regressed against slope values (**σ**) of the same linear model to obtain a visual cue of the water use space. Values transition from more (**purple**) to less (**red**) conservative.

We then plotted the intercept estimated by the linear model for droughted trees against its slope “σ”, which allowed us to observe a phylogenetic signal across species and families along an water use spectrum (Figure 5) that is consistent with earlier work (Brodribb *et al*., 2014) (Figure 4 and Supplementary table 10). The derived Cuprassaceae *C. lawsoniana* sits at awater spending extreme followed by the derived Cupressaceae *J. communis* and Taxaceae *T. baccata*, and by the Pinaceae *P. menziesii*. The basal Cupressaceae *S. sempervirens*, and the Pinaceae *P. sitchensis* and *P. sitchensis x P. glauca* occupied a more conservative portion of the spectrum, followed by other Pinaceae, *L. eurolepis* and *T. heterophylla*.

### Accumulation of ABA in response to drought

**Figure 6:**
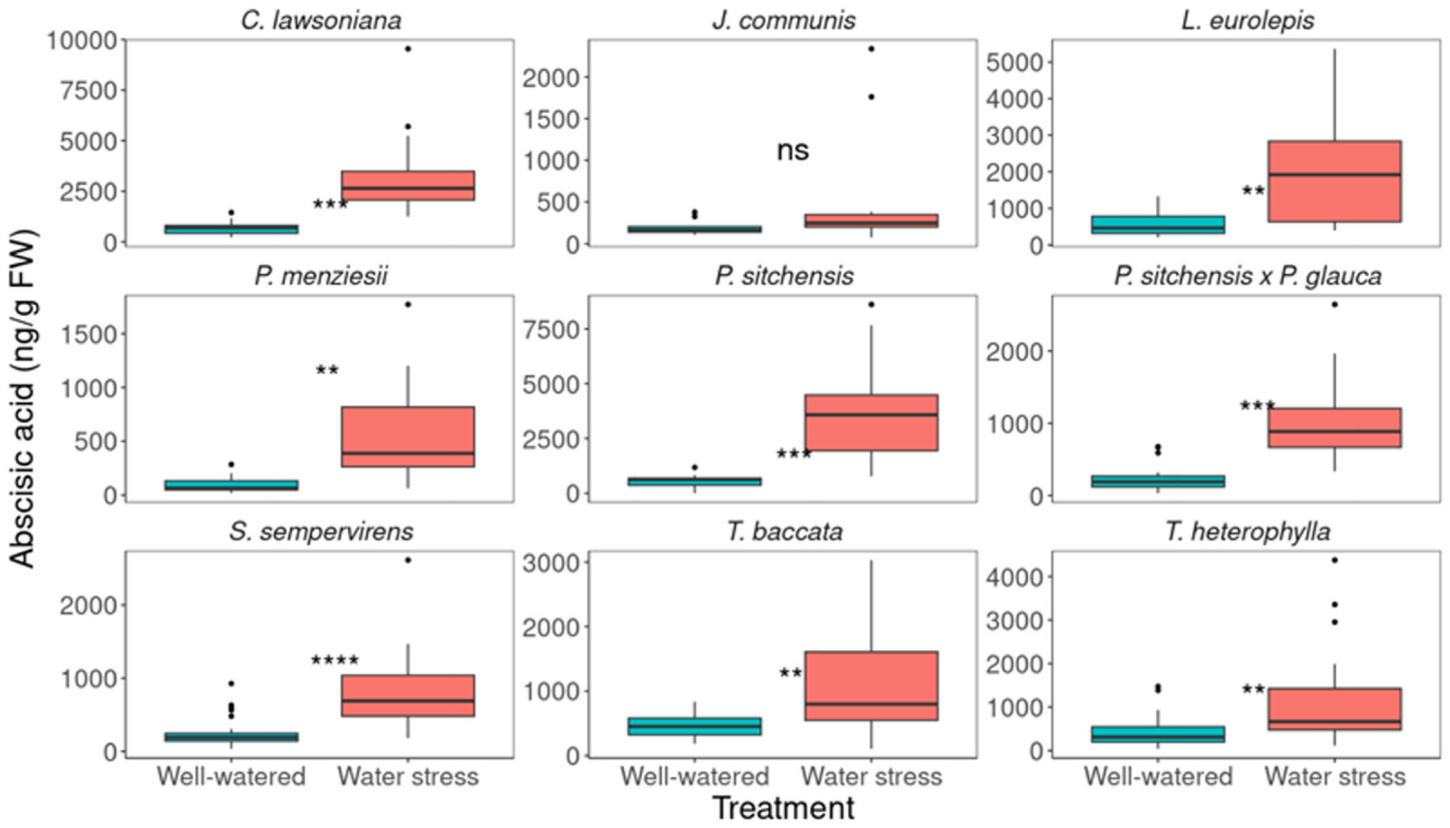
Abscisic acid levels across drought treatments. **Blue**: drought-treated trees, N = 16-32; **Red**: well-watered control trees, N = 16-32. **Asterisks** represent significant differences between treated and control trees; **Boxes** indicate median, 25% and 75% quantiles. **Bars** indicate standard deviation. **Dots** represent potential outliers; Student’s t-test (P value < 0.05).

**Figure 7:**
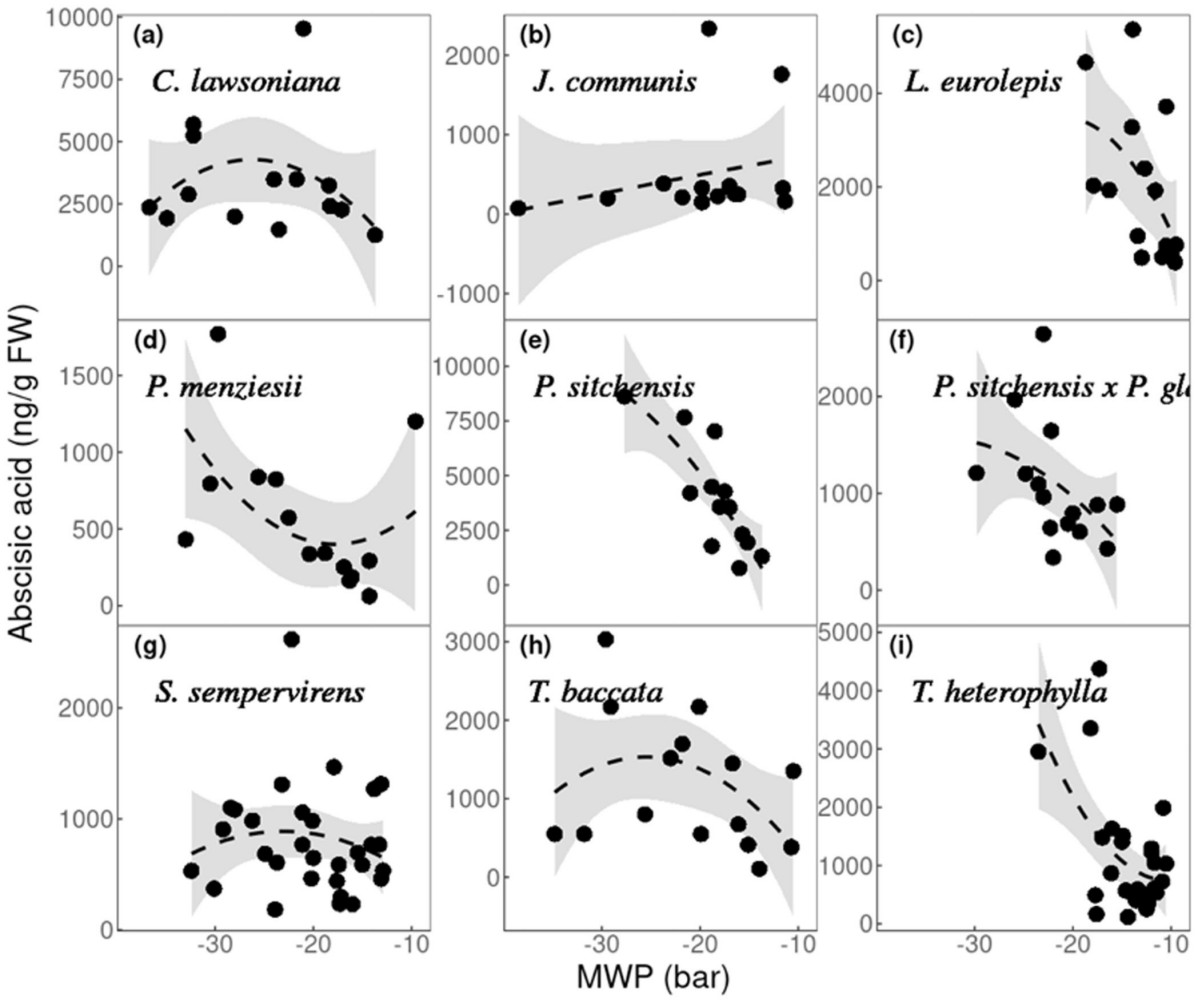
Abscisic acid accumulation in relation to MWP. A second order polynomial function was fit to the data (**dashed line**). Species showing decreasing ABA levels as MWP became more negative were considered Peaking types, and species with increasing ABA levels as MWP became more negative were considered Rising type. N = 16-32.

The level of ABA was determined in all species at 4-5 time points. Large differences in ABA levels developed between well-watered controls and droughted plants, except in *J. communis* (Figure 6). The data also indicate very different accumulation profiles among species relative to MWP (Figure 7). In *C. lawsoniana*, high ABA levels accumulated after a few days of treatment, peaking at nearly 10,000 ng of ABA and then returning to levels close to 2000 ng despite MWP becoming more negative. This ABA profile in *C. lawsoniana* is similar to a P-type as described (Brodribb *et al*., 2014). *J. communis* followed a similar profile but had a less pronounced peak at ~2000 ng of ABA and overall, it had low levels of ABA. *T. baccata* showed an ABA profile suggestive of a P-type, reaching as high as 3000 ng of ABA and then decreasing slowly to ~500 ng. The basal Cupressaceae *S. sempervirens* had an unclear ABA profile, not rising enough to be clearly assigned to a peaking or rising type.

In contrast, droughted Pinaceae plants accumulated ABA throughout the drought, reaching or approaching a plateau without decreasing, with each species varying in ABA levels. Several species had ABA levels in the range of 1000 ng to 2000 ng, with the exceptions of *L. eurolepis* (∼5000 ng) and remarkably high levels were found in *P. sitchensis* (∼9,000 ng). Their profiles were consistent with an R-type (Brodribb *et al*., 2014).

Based on the observation ABA response matching P-type and R-type profiles, gene expression investigations in ABA related genes were conducted in C. *lawsoniana, P. sitchensis* and *T. baccata* with the aim of encompassing a range of responses and taxa.

### Gene expression

**Figure 8:**
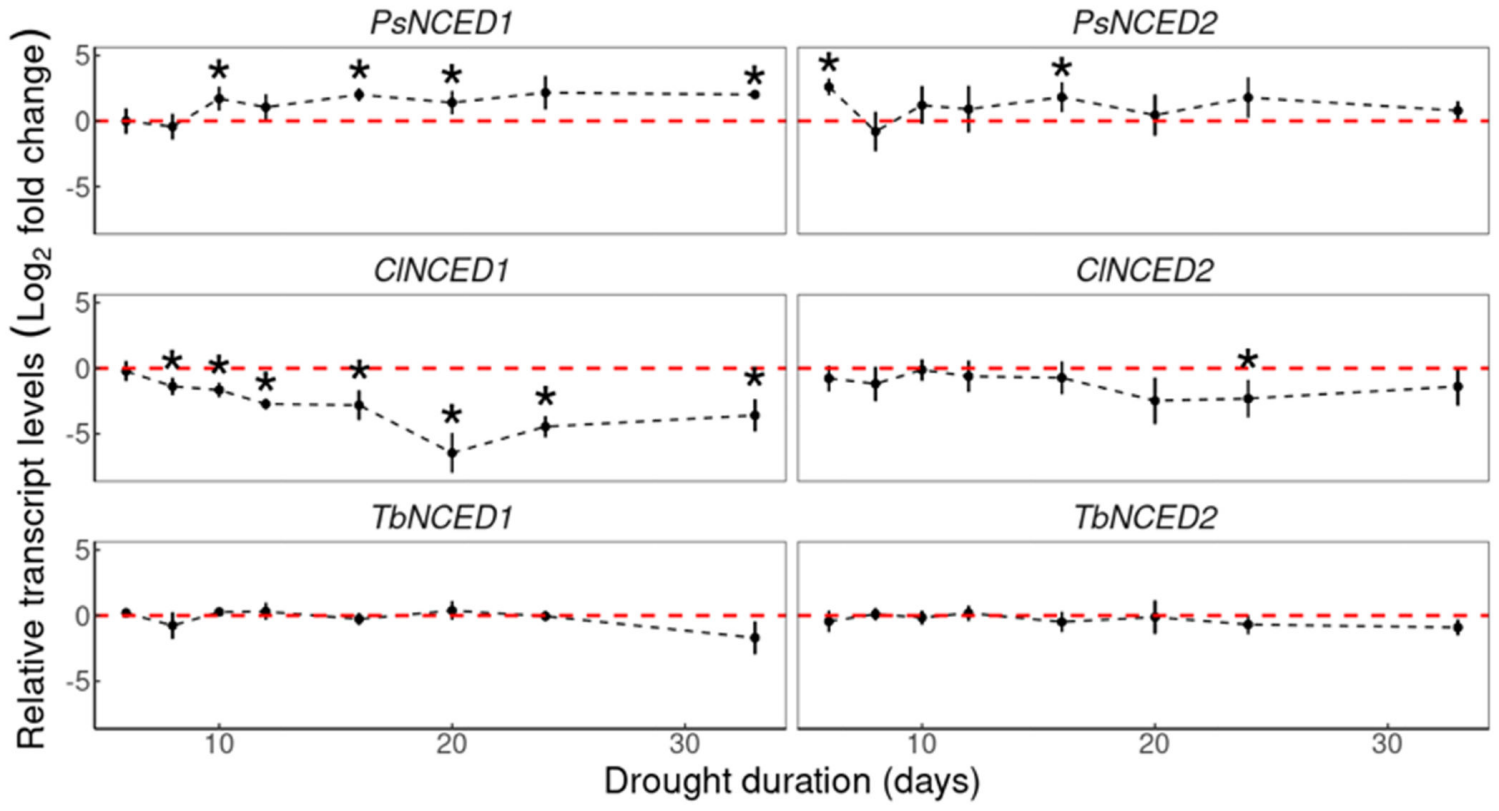
Relative transcript levels at 8 time points during the imposed drought stress for each of the putative ortholog NCED sequences identified. Raw Ct values were normalised against well-watered controls and reference genes to obtain ΔΔCt values. **Asterisks** represent significant differences between treated trees and the zero-fold-change baseline (**dashed red line**); Tukey post-hoc test (adjusted P value < 0.05). N = 3-4.

Expression of the *nced* sequences identified was analysed in foliage samples from trial 1 by RT-qPCR with gene-specific primers. The data showed a 2 to 3 Log_2_ fold increase in *nced* gene expression in *P. sitchensis*, starting from 10 days of drought (Figure 8). The increase was more consistently detected for *PsNCED1* than for *PsNCED2*. In contrast, the *C. lawsoniana* sequence *ClNCED1* was clearly down-regulated from 8 days of drought, decreasing by as much as a -7 Log2 fold change). *ClNCED2* expression decreased later and more variably from 20 to 33 days. There was no significant differential expression for *T. baccata nced* genes.

**Figure 9:**
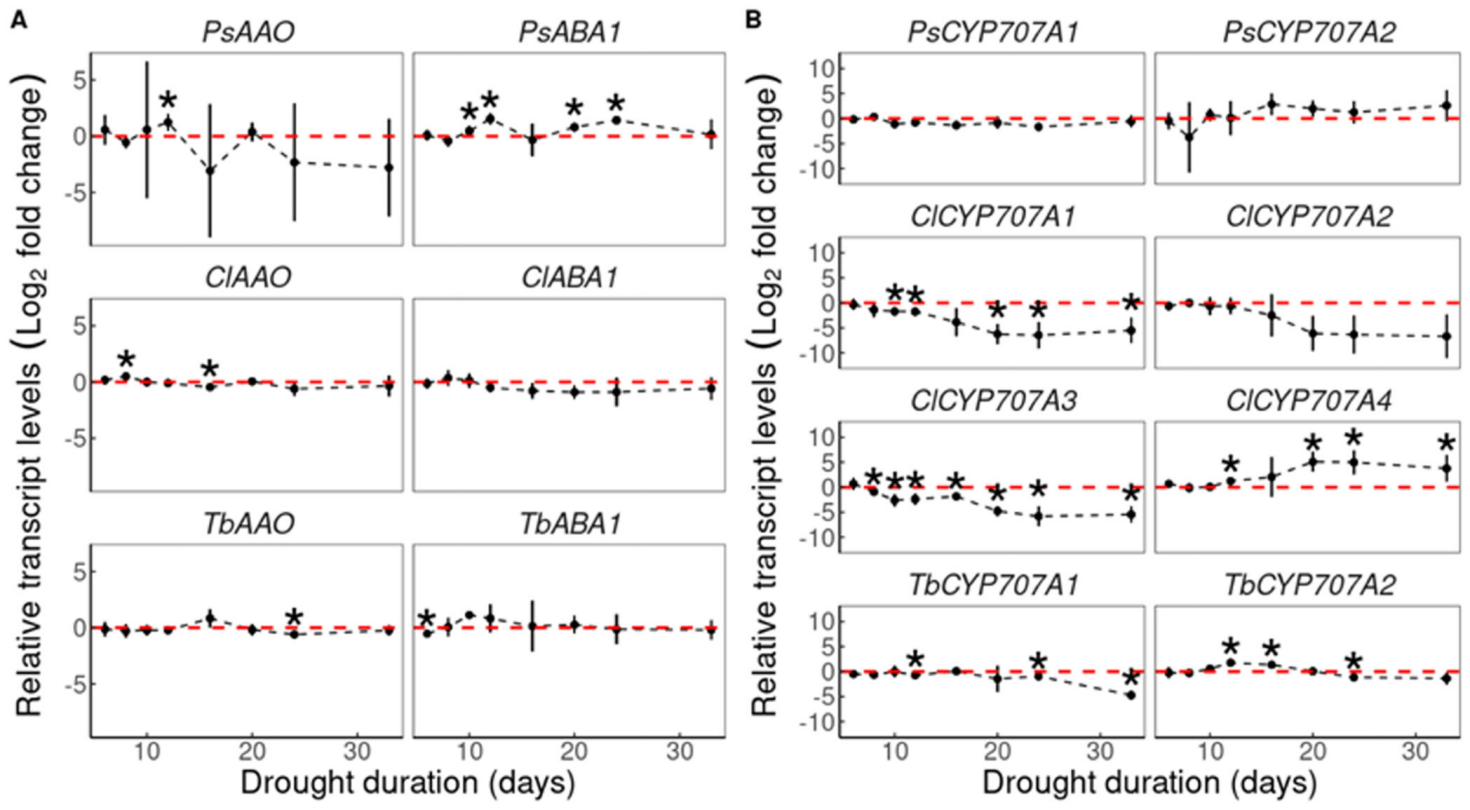
Relative transcript levels at 8 time points during the imposed drought stress for each of the putative ortholog ABA1 and AAO (**A**) and CYP707A (**B**) sequences identified. Raw Ct values were normalised against well-watered controls and reference genes to obtain ΔΔCt values. **Asterisks** represent significant differences between treated and the zero-fold-change baseline (**dashed red line**); Tukey HSD post-hoc test (adjusted P value < 0.05). N = 3-4.

Expression of a single sequence for both *AAO3* and *ABA1* involved in ABA biosynthesis was analysed in the same set of samples. In *P. sitchensis, PsABA1* was significantly overexpressed relative to controls at 12, 20 and 24 days of drought, showing a 1 to 2 Log2 fold increase in transcripts abundance. *PsAAO* was significantly overexpressed at about the same level but only at 12 days of drought and decreased afterwards producing variable and non-significant results (Figure 9A). *ClAAO* was weakly but significantly overexpressed at eight days but downregulated at 16 days of drought. *ClABA1* appeared to follow the same pattern as *ClAAO* but the changes were non-significant (Figure 9A). In *T. baccata*, the data indicated variable levels of gene expression, with weak but significant down-regulation of *TbAAO* only at 24 days and *TbABA1* at 33 days of drought, while no clear signal was detected for other time points.

We also investigated the expression of putative catabolic *CYP707A* genes, responsible for the breakdown of ABA (Figure 9B) (See Supplementary material for sequence analysis details). There was no significant change in the expression of *PsCYP707A1s* in *P. sitchensis*. In contrast, the *C. lawsoniana ClCYP707A4* was significantly overexpressed up to 6Log_2_ fold starting from 12 days of drought stress, but *ClCYP707A1* and *ClCYP707A3* were strongly downregulated from 10 and 8 days of drought respectively, the lowest values reaching a 6 to 7 Log_2_ fold decrease, and *ClCYP707A2* did not change significantly. In *T. baccata TbCYP707A1* expression was significantly downregulated at three time points (12, 24 and 33 days of drought) with its lowest mean negative fold change at 33 days of drought stress (2 to 3 Log_2_ fold change). *TbCYP707A2* appeared to vary its expression from significantly up- to down-regulated between the second week and third week of drought, being weakly differentially expressed at 12, 16 and 24 days.

## Discussion

In this study, we systematically analysed several conifer transcriptomes to uncover gene sequences in ABA metabolism and explored if their expression responsive to drought may contribute to characteristic ABA profiles. We identified an *nced* gene that is upregulated in response to drought, suggesting it is involved in ABA biosynthesis in *P. sitchensis*, which has a characteristic R-type ABA profile. In contrast, *nced* genes were downregulated and a candidate ABA catabolism gene was upregulated in *C. Lawsoniana*, which had a clear P-type profile. By tracking changes in the midday leaf water potential (MWP) in droughted trees across different conifer species, we observed a range of responses from conservative to more permissive strategies, along with a range of ABA response profiles. The Cupressaceae *C. lawsoniana, J. communis* and *S. sempervirens* as well as the Taxaceae *T. baccata* reached a more negative shoot water potential under drought stress, while the change in MWP in the Pinaceae was either small as in *L. eurolepis* and *T. heterophylla* or non-significant as in *P. sitchensis*. We discuss how findings relating to gene family analyses and drought responsive expression may inform our understanding of ABA profile regulation and adaptation to drought, as well as practical applications across conifer tree species.

### Water relations and ABA profiles under drought conditions

We used volumetric SWC to model the response of MWP to drought stress as described elsewhere (Attia *et al*., 2015; Bayar and Deligöz, 2020). Volumetric SWC was an overall good predictor of shoot MWP and allowed us to classify species along the water use spectrum as in previous work (Martinez-Vilalta *et al*., 2014). We used the slope (σ) of the drought-stress regression line to rank plants as follows: *L. eurolepis* and *T. heterophylla* as strict conservative; *P. sitchensis, P. sitchensis x P. glauca* and *S. sempervirens* as relative conservative; *P. menziesii, J. communis* and *T. baccata* as more permissive; and *C. lawsonia*na as extreme permissive.

By plotting the coordinates of the intercept and slope of the linear regression between MWP and SWC a further distinction between derived and basal Cupressaceae was apparent, with *S. sempervirens* – basal clade – clustering together with the more conservative Pinaceae. These results are consistent with studies on the adaptive significance of variation in water relations across the conifer phylogeny and on resistance to embolism (Jacobsen *et al*., 2007; Willson, Manos and Jackson, 2008; Choat *et al*., 2012; Brodribb *et al*., 2014; Bayar and Deligöz, 2020).

We obtained drought-responsive ABA accumulation profiles ranging from Rising (R-) to Peaking (P-) types (Brodribb and McAdam, 2013; Brodribb *et al*., 2014). The ABA profiles found in our study were in general agreement with previous reports of P-type profiles found in *C. laswoniana* and *J. communis* and *T. baccata*.

We were able to clearly classify all Pinaceae tested as R-types. This is consistent with a strict conservative response characterised by continuous accumulation of ABA to prevent excessive water loss through stomatal leakage and to avoid embolism. We found that P-profiles in *J. communis* and *T. baccata* were less obvious, and this was likely due to high tree-to-tree variation in ABA levels and the relatively short drought treatment. The basal Cupressaceae *S. sempervirens* eluded our classification, as it did not clearly display a rising nor a peaking accumulation profile.

### Drought-responsive expression of NCEDs and ABA accumulation

In conifers, most gene expression studies on drought responses have either analysed transcriptome changes (Haas *et al*. 2021, Du *et al*. 2018, Fox *et al*. 2017) or focused on defence gene families such as dehydrins (Baldi et al. 2022, Stival Sena et al. 2018, Moran et al. 2017) and NLR (van Ghelder 2019). The expression of ABA metabolism genes was shown to be drought responsive in *Picea abies* and *Pinus sylvestris (Pashkovskiy et al. 2019)*, but the study gave few insights into their molecular evolution or their role in functional diversity across conifers as both species are in the Pinaceae. Our sequence similarity searches identified candidate ABA-related genes by mining new and existing transcriptomes from a range of conifer species. While genes involved in ABA accumulation may be conserved across the land plant phylogeny (Sussmilch, Brodribb and Scott A.M. McAdam, 2017; McAdam and Sussmilch, 2021), our observations suggest that their regulation in conifers may differ from that reported in model angiosperms. We identified two sequence clusters of putative *NCED* sequences across several conifer species, which we named *NCED1* and *NCED2*. These sequences were highly conserved, and formed two sub-groups that were both sister to angiosperm *NCEDs*. We observed a range of drought responsive *NCED* expression profiles when comparing conifer species from different families.

In *P. sitchensis* (Pinaceae), *PsNCED1* expression increased starting from day 10 of the drought treatment and remained elevated relative to the controls, confirming previous evidence from Pashkovskiy *et al*. 2019, who observed a correlation between ABA levels and NCED expression in spruce needles. By comparison, *PsNCED2* was more variable with significant overexpression at only two time points and especially after 6 days of drought, before changes in *PsNCED1* were detected. These observations are consistent with the timing of ABA accumulation during drought in *P. sitchensis*. The transcript levels of *PsNCED1* and *PsNCED2* suggest that they may have partly complementary roles in ABA accumulation. Based on our data, *PsNCED1* could be the main biosynthetic gene controlling ABA accumulation in *P. sitchensis* and, as such, it may underpin the ABA R-type we observed.

Our observations in *C. lawsoniana* indicated that P-type response may involve the downregulation of *NCED* expression coinciding with a drop in ABA. *ClNCED1* was clearly and progressively downregulated. We could not establish a clear pattern for the group 2 sequence *ClNCED2*. We propose that *ClNCED1* is a candidate rate-limiting enzyme in *C. lawsoniana*. Downregulation happened before the peak of ABA was observed during the first two weeks of drought, which could be explained by a lag between changes in transcript levels and enzyme activity, or by the sampling time points. *ClNCED2* may be weakly responsive to drought stress or may be active in other processes and tissues. In contrast, in *T. baccata* we did not detect any differential expression *NCED* genes despite increased levels of ABA in response to drought.

In summary, we confirmed that an R-type conifer upregulated *NCED* genes expression, which is a long-term response that may support ABA *de novo* biosynthesis for the duration of the drought event. On the contrary, we did not find evidence of upregulation of *NCEDs* in a P-type conifer. This finding contrasts with previous research in other plant species, where *NCEDs* have always been found to be upregulated during drought responses (Burbidge et al. 1997, Pashkovskiy et al. 2019, Lee et al 2021). A pioneering study suggested that P-type profiles in embolism resistant conifers are based on inhibition of ABA biosynthesis in the leaf (Mercado-Reyes et al 2023). We here have provided molecular evidence for this mechanism, based on continuous downregulation of *NCEDs*, which may complement ABA removal but does not correspond to the peak in foliage ABA levels. They also observed that removal of ABA from the foliage occurred by conjugation into ABA-GE stores - not catabolism - a process that we did not investigate in our study. These observations may indicate an evolutionary differentiation in gene expression pathways and ABA accumulation mechanisms among conifer families, opening to future research on the molecular bases of P-type ABA profiles.

### Drought-responsive expression of other candidate ABA metabolism genes

We explored *ABA1* and *AAO* sequences and found a high degree of similarity between taxa, suggesting that these genes are highly conserved across the land plants phylogeny. *ABA1* and *AAO3* can support ABA biosynthesis at lower expression levels compared to key *NCEDs* under drought stress in model plants (Marin *et al*., 1996; Audran *et al*., 1998; Seo *et al*., 2000b; Koiwai *et al*., 2004; Barrero *et al*., 2006). In conifers, we found that *AAO* and *ABA1* only had minor changes in expression, with a few exceptions; similar results were reported in *P. abies* and *P. sylvestris* (Pashkovskiy *et al*. 2019). The minor upregulation of *PsABA1* in *P. sitchensis* may complement the strong upregulation of *PsNCED1* and thus support ABA accumulation. In *C. lawsoniana, ClABA1* and *ClAAO* expression results suggest a potential contribution to peaking ABA profile as they switched from weakly upregulated to weakly downregulated. Taken together, our observations suggest that changes in *AAO* and *ABA1* expression may have a minor role in driving changes in ABA levels.

In *A. thaliana, CYP707A1-A4* genes encode 8′-hydroxylases have a role in controlling cellular ABA levels through catabolic oxidation of ABA to phaseic acid and are upregulated upon re-hydration (Kushiro *et al*., 2004; Saito *et al*., 2004; Okamoto *et al*., 2009). We identified two groups of conifer *CYP707A*s. Group 1 included sequences that were more closely related to *A. thaliana CYP707A1* and *CYP707A3*, known to function in leaf drought responses (Okamoto *et al*., 2009). Group 2 was more closely related to *A. thaliana CYP707A2*, a gene that is involved in seed dormancy in plants (Kushiro *et al*., 2004; Matakiadis *et al*., 2009; Zhu *et al*., 2011). The Group 2 sequences were more diversified with multiple putative paralogs in *C. lawsoniana* and *L. laricina*. In *C. lawsoniana*, our expression results suggest that the four *CYP707A* could act together to modulate ABA levels. Under this scenario, the downregulation we observed in *ClCYP707A1, 2* and *3* at the early stages of drought could contribute to rising ABA levels due to reduced catabolism. On the other hand, upregulation of *ClCYP707A4* may counteract this process and remove ABA starting from the second week of drought to produce the typical peaking type we have observed. As mentioned above, other studies suggest that P-types are less likely to result from ABA degradation to PA than from conjugation to form ABA-GE (Mercado-Reyes *et al*, 2023). We didn’t find any significant change in *P. sitchensis* sequences *PsCYP707A1* and *PsCYP707A2*, meaning that the ABA catabolic function is either maintained at constitutive levels or not required. This supports our interpretation of a key role for *PsNCED1* in driving the R-type ABA profile observed in *P. sitchensis*. In *T*.

*baccata* the only genes that were differentially expressed were *TbCYP707A*s. We observed that *TbCYP707A1* was weakly but significantly downregulated. In contrast, *TbCYP707A2* switched from up to downregulation, suggesting that *TbCYP707As* may be downregulated to allow ABA accumulation. The functional characterization of *CYP707As* and other genes related to ABA-GE may be a fruitful area of research in the future. In particular, further work is needed to elucidate the molecular mechanisms of ABA accumulation and removal in species such as *C. lawsoniana* and *T. baccata*.

### Practical applications for the sustainable use of conifer trees

Trees are being affected by more frequent and intense drought events globally (Allen *et al*., 2010; McDowell *et al*., 2016) and conifer trees are most vulnerable to these ongoing environmental changes (Farjon 2018). An improved understanding of drought physiology including the molecular mechanisms investigated here may inform practical applications for sustainable use of conifers in different ways. First, species with different responses to water deficit may be matched to changing conditions based on climate trends, such as the duration, intensity and frequency of droughts. Our study contributes to the body of knowledges on the differences between Pinaceae and derived Cupressaceae (Brodribb *et al*., 2014; Harfouche, Meilan and Altman, 2014). This understanding indicates that species such as spruces (Pinaceae) respond by closing their stomata more tightly which decrease their productivity during episodic droughts, making them less suitable for dry sites, compared to derived Cupressaceae such as *Chamaecyparis* (Harfouche, Meilan and Altman, 2014). Similarly, forest management such as soil draining and stand thinning, which may lower or raise the water table, respectively, will affect these species groups differently (Savill et al. 1997, chapters 5, 6, 10). Second, genes that regulate drought responses such as *NCEDs* may become the targets for molecular breeding approaches (Grattapaglia et al. 2018). Further research could assess whether DNA sequence variations in these genes impact upon traits such as water use efficiency or biomass accumulation under drought, allowing for selection in tree breeding programmes, including development of hybrids with desirable allele combinations (Baldi et al. 2016). Third, bioengineering approaches may be developed (Rodriguez et al. 2022) to alter the ABA response profiles or alternatively to increase the expression of drought protective genes such as dehydrins (Stival Sena et al. 2018).

## Conclusions

“This work explored the molecular mechanisms of conifer ABA drought responses, which are poorly studied. The genes of interest participate in the abscisic acid (ABA) biosynthesis pathway and appeared conserved across millions of years of evolution. Although not definitive, we bring new evidence that 9-cis-epoxycarotenoid dioxygenases (NCEDs) fine-tune ABA accumulation in conifer foliage in two diverging modes, depending on the phylogenetic history. Our experiment adds to the growing body of evidence suggesting that R/P types of ABA accumulation may be an evolutionary trait that emerged during conifer diversification and adaptation to dry environments. Functional analysis of the *NCED1* gene products may be a fruitful area of future study. Further work is needed to assess the origin of the ABA peak that we observed in *C. lawsoniana*. ABA-GE mobilisation is catalysed by β-glucosidases and future studies should account for their expression. From an applied perspective, our findings on the molecular basis of ABA responses to drought across species may aid the identification or development of trees able to perform better under abiotic stress.”

## Supplementary Material

Supplementary material

## Figures and Tables

**Table 1:** Summary table of drought trials. **Trial**: first, second and third; **Species**: conifer species tested in each of the three trials; **Duration**: number of days of experimental drought; **Time points**: number of time points for sample collection in each trial; **Sampling days**: time points for sample collection since water withheld.

Trial	Species	Duration (days)	Time points	Sampling days
1	*C. lawsoniana*, *J. communis*, *P. sitchensis*, *P. sitchensis x P. glauca,* *T. baccata*	34	8	6, 8, 10, 12, 16, 20, 24, 33
2	*C. lawsoniana*, *P. sitchensis*,	25	4	6, 9, 16, 24
	*P. sitchensi x P. glauca,* *T. baccata,* *L. eurolepis,* *P. menziesii*			
3	*T. heterophylla,* *S. sempervirens*	35	5	6, 12, 18, 26, 34

## Data Availability

The raw RNA sequences, transcriptomes, and sample meta data supporting this publication can be publicly accessed in NCBI GenBank under the BioProject PRJNA421925. All BioSamples, Sequence Read Archive (SRA), and Transcriptome Shotgun Assembly (TSA) accessions are linked to this BioProject. Datasets for physiological, ABA and gene expression analyses are available at the following link: https://doi.org/10.5281/zenodo.7870477 Protein sequence alignments are available at the following link: https://doi.org/10.5281/zenodo.7807814
