# Supplementary material for: Contrasted NCED gene expression across conifers with Rising and Peaking abscisic acid responses to drought
Source: Plant Stress. Author manuscript; Available in PMC 2025 Mar 19. (PMC11913745; doi:10.1016/j.stress.2024.100574)
Supplement: Supplementary material [file EMS203899-supplement-Supplementary_material.pdf]

Supplementary material

I. Previous drought experiment and transcriptome work

Plant materials

The study used two-year old saplings of six different conifer species belonging to the Pinaceae, Taxaceae and Cupressaceae (see Table S1 below). The saplings were obtained from Alba Trees Nurseries (Lower, Winton Way, Tranent EH33 2AL) as dormant plants ranging from 30 to 60 cm in height; the saplings were transferred to 3-L pots containing a 3:1 ratio general potting mix (M2, ICL, Everris ltd, 4190 CA Geldermalsen, The Netherlands) and grit sand (Bathgate horticulture, Vale Park, Evesham, Worcestershire WR11 1GP). The potted saplings were placed in a greenhouse for six weeks to initiate growth with a temperature of 18 °C (night-time low) to 25 °C (daytime high), with supplemental lighting to achieve a photoperiod of 16:8 hours (Day:Night), watering every 2 to 3 days and addition of N-P-K 20:20:20 fertilizer (Chempak, Suffolk, United Kingdom) after four weeks.

Drought treatment and tissue sampling

A drought experiment was run with a water-stress (WS) treatment and well-watered controls (WW) using a randomised split plot design with two complete blocks. For the WS saplings, all water was withheld until the water content dropped to 20% of field capacity. The control plants were watered every 2-3 days and their field capacity was close to 100% throughout the experiment. We monitored the water in the pots by weighing the plants at regular intervals to estimate the water availability in 33% of pots. A total of 48 saplings per species were divided equally into WW (24 plants per species) and WS (24 plants per species) and distributed evenly in the two complete blocks (12 WW and 12 WS plants per species), using the same greenhouse conditions as above.

Tissue samples from six randomly selected WW saplings and six randomly selected WS saplings were collected after 14 days of drought, except in CLA where it was at 12 days, when the WS plants had reached 20% of water capacity on average. The tissues sampled were young foliage (YL), primary stem (PS), stem secondary xylem (SX), stem bark and phelloderm (SB), root secondary xylem (RX), root bark and phellogen (RB) as described in (Duval *et al.*, 2014). All tissues were immediately flash-frozen in liquid nitrogen and stored at –80 °C until further use.

RNA isolation and sequencing

Total RNA was isolated from tissue ground in liquid nitrogen to a fine powder by using cetyltrimethyl ammonium bromide (CTAB) extraction method as described by (Chang, Puryear and Cairney, 1993) with modifications (Pavy *et al.*, 2008). The total RNA concentration was determined using a ND-1000 UV-Vis Spectrophotometer (ThermoFisher Scientific, Massachusetts, United States); the RNA quality with an Agilent 2100 Bioanalyzer and Agilent RNA 6000 Nano Kit LabChips (Agilent, California, United States), and stored at −80°C. All samples used in the study had an Agilent 2100 Bioanalyzer RIN score of 6.0 to 8.0. The RNA samples were used for: 1) reference transcriptome development by creating a single equimolar pool of RNAs for each of the six species by combining RNA all tissues and both WS and WW saplings; 2) gene expression analysis by using individual samples of both WW and WS saplings including YL, SX, and SB tissues of *C. lawsoniana*, *P. sitchensis*, and *T. baccata*, with five biological replicates per treatment, tissue type and species (73 samples in total, see Table S2). The RNA-sequencing was carried out in different core facilities for the reference transcriptome and the gene expression study.

Library construction and sequencing for the reference transcriptome development were conducted at McGill University and Genome Quebec Innovation Centre. Total RNA was quantified using a NanoDrop Spectrophotometer ND-1000 (NanoDrop Technologies, Inc.) and its integrity was assessed using a 2100 Bioanalyzer (Agilent Technologies). Libraries were generated from 250 ng of total RNA using the TruSeq stranded mRNA Sample Preparation Kit (Illumina), as per the manufacturer's recommendations. Libraries were quantified using the Quant-iTT PicoGreenR dsDNA Assay Kit (Life Technologies) and the Kapa Illumina GA with Revised Primers-SYBR Fast Universal kit (Kapa Biosystems). Average size fragment was determined using a LabChip GX (PerkinElmer) instrument. The libraries were normalized, denatured in 0.05N NaOH and then were diluted to 7pM using HT1 buffer. The clustering was done on an Illumina cBot and the flowcell was ran on a HiSeq 2500 for 2x250 cycles in rapid mode following the manufacturer's instructions.

Library construction and sequencing for the transcriptome profiling study were done at High Throughput Genomics service at Oxford Genomics Centre. PolyA enriched, directional library was prepared using Illumina TruSeq Stranded mRNA kit and using standard universal Illumina multiplexing adapters, followed by in-house indexing primers during amplification (unique dual indexing). Sequencing was performed using the HiSeq4000 with paired-end 75bp reads.

The adapter sequences were removed using Cutadapt 1.9.1 (Martin, 2011) and the reads were trimmed and filtered using PRINSEQ 0.20.4 [51]. The quality of reads was checked through FastQC 0.11.5 (*FastQC: a quality control tool for high throughput sequence data – ScienceOpen*, no date) to ensure that high-quality reads were obtained. The transcripts were assembled using Trinity 2.4.0 (Haas *et al.*, 2013) and only the longest isoforms were chosen to represent their corresponding genes. Gene level abundances were quantified through RSEM (Li and Dewey, 2011) and genes with FPKM>=1 were retained for further analysis. Blastx (Camacho *et al.*, 2009) was run for gene sequences against a database composed of gymnosperms, angiosperms, animals, fungi, and bacteria proteins from PLAZA (Van Bel *et al.*, 2022), Ensembl (Howe *et al.*, 2021) and Ensembl Genomes (Howe *et al.*, 2020) at the parameter "-evalue 1e-5" and "-max_target_seqs 1", and only sequences with the best hit on a plant protein were considered for inclusion in the reference transcriptome. Further filtering involves the identification and removal of non-Viridiplantae genes via blastn against BLAST nucleotide sequence (nt) database and contaminated sequences via blastn against the UniVec Database (https://ftp.ncbi.nlm.nih.gov/pub/UniVec/). We benchmarked the transcripts in the assemblies using BUSCO v5.1.2 (Manni *et al.*, 2021) and OrthoDB v10 (Kriventseva *et al.*, 2019) embryophyta database to evaluate the assembly completeness.

The transcriptome assemblies across the six species had between 30,003 and 70,639 transcripts (see Table S3 for assembly statistics). The BUSCO assessment confirmed that the majority of embryophyte core genes were present in these transcriptomes, with over 80% of complete BUSCOs (single-copy or duplicated) recovered in all the assemblies.

Supplementary table 1A. Species information and BioSample accession numbers in the Short Read Archive (SRA).

**Species**	**Family**	**SRA Accession**
Chamaecyparis lawsoniana (A. Murray bis) Parl.	Cupressaceae	SAMN08384894
Picea sitchensis (Bong.) Carr.	Pinaceae	SAMN08384897
Taxus baccata L.	Taxaceae	SAMN08384899

Supplementary table 1B. Number of biological replicates per **Species** (**CLA**, *Chamaecyparis lawsoniana*; **PSI**, *Picea sitchensis*; **TBA**, *Taxus baccata*), **Treatment** (Well-watered, **WW**; Water-stressed, **WS**) and **Tissue** (**YL**, Young foliage; **SX**, Secondry Xylem; **SB**, Stem Bark).

**Species**	**WW**			**WS**		
	**YL**	**SX**	**SB**	**YL**	**SX**	**SB**
**CLA**	5	5	5	5	5	5
**PSI**	4	4	4	3	3	3
**TBA**	5	5	5	2	3	2

Supplementary table 2. Summary of transcriptome assembly statistics of the three conifer species.

	C. lawsoniana	*P. sitchensis*	*T. baccata*
Abbreviation	CLAR	PSIR	TBAR
Number of transcripts	30,003	41,861	41,425
Total length of transcripts (bp)	41,908,465	42,053,211	46,114,685
Average transcript length	1,396.8	1,004.6	1,113.2
N50	2,096	1,700	1,947
BUSCO score (N = 1,614)	S:82.3, D:3.0, F:4.7, M:10.0	S:78.4, D:2.6, F:8.6, M:10.4	S:80.8, D:3.7, F:5.0, M:10.5

Literature cited in this section

Camacho C, Coulouris G, Avagyan V et al. 2009. BLAST+: Architecture and applications. *BMC Bioinformatics* 10: 1–9.

Chang S, Puryear J and Cairney J. 1993. A simple and efficient method for isolating RNA from pine trees. *Plant Molecular Biology Reporter* 11:113–116.

Duval I, Lachance D, Giguère I, Bomal C, Morency MJ, Pelletier G, Boyle B, MacKay JJ, Séguin A. 2014. Large-scale screening of transcription factor-promoter interactions in spruce reveals a transcriptional network involved in vascular development. *Journal of Experimental Botany* 65:2319–2333.

Andrews S. 2010. FastQC: a quality control tool for high throughput sequence data – *Babraham Bioinformatics*. Available at: https://www.scienceopen.com/document?vid=de674375-ab83-4595-afa9-4c8aa9e4e736

Haas B, Papanicolaou A, Yassour M et al. 2013. De novo transcript sequence reconstruction from RNA-seq using the Trinity platform for reference generation and analysis. *Nature protocols* 8:1494–1512.

Howe KL et al. 2020. Ensembl Genomes 2020—enabling non-vertebrate genomic research. *Nucleic Acids Research* 48:D689–D695.

Howe KL et al. 2021. Ensembl 2021. *Nucleic acids research* 49:D884–D891.

Kriventseva EV, Kuznetsov D, Tegenfeldt F, Manni M, Dias R, Simão FA, Zdobnov EM. 2019. OrthoDB v10: sampling the diversity of animal, plant, fungal, protist, bacterial and viral genomes for evolutionary and functional annotations of orthologs. *Nucleic acids research* 47:D807–D811.

Li B and Dewey CN. 2011. RSEM: Accurate transcript quantification from RNA-Seq data with or without a reference genome. *BMC Bioinformatics* 12:1–16.

Manni M, Berkeley MR, Seppey M, Simão FA, Zdobnov EM. 2021. BUSCO Update: Novel and Streamlined Workflows along with Broader and Deeper Phylogenetic Coverage for Scoring of Eukaryotic, Prokaryotic, and Viral Genomes. *Molecular Biology and Evolution* 38:4647–4654.

Martin M. 2011. Cutadapt removes adapter sequences from high-throughput sequencing reads. *EMBnet.journal* 17:10–12.

II. Drought physiology, ABA, phylogenetic analysis, and gene expression

Tables

Supplementary table 3. Candidate gene (**Conifer ID**), with relative forward and reverse primers (**Forward** and **Reverse sequence**) reference gene (**Model Name** and **Model ID**) and housekeeping (bold characters) gene sequence and primer information.

**Model Name**	**Model ID**	**Conifer ID**	**Forward sequence**	**Reverse sequence**	**Gene**
NCED	AT3G14440	PSIR_DN33063_c0_g1	cagatcccggccttcacac	ggggatactcctcggcatc	9-cis-epoxycarotenoid[illegible] dioxygenase
NCED	AT3G14440	PSIR_DN39298_c0_g1	cgtcgtaggcataggggttc	tcggaggacacaccatttg	9-cis-epoxycarotenoid[illegible] dioxygenase
CYP707A3	AT5G45340	PSIR_DN32321_c0_g1	tgttggtaggaagcacgatacac	tcctcaatcaccttcggttatg	Cytochrome P4[illegible] family 707
CYP707A3	AT5G45340	PSIR_DN32134_c0_g1	aacgttaataatggccctggtg	tcctgaatgatcacgacaactc	Cytochrome P4[illegible] family 707
AAO3	AT2G27150	PSIR_DN38520_c0_g1	gtgcgagacatgccgtaag	gccacactttgcctgtaagc	aldehyde oxidas[illegible]
ABA1	AT5G67030	PSIR_DN41411_c0_g1	tgcgtacagaggtccaatcc	cgccagtaatgcaaccattctcc	zeaxanthin epoxidase (ZEP[illegible]
mRNA capping enzyme family protein	AT3G09100	PSIR_DN38225_c0_g2	tttccttgtcagctgtgatgc	ttccattcgtaccagcatcc	**HOUSEKEEPI[illegible]**
NCED	AT3G14440	CLAR_DN53708_c0_g1	gcaaatgcacaaggattgc	aagcaacggattggactcg	9-cis-epoxycarotenoid[illegible] dioxygenase
NCED	AT3G14440	CLAR_DN53708_c0_g5	ggcgaggatgaggagattg	cttcttttgctctctcctgtcg	9-cis-epoxycarotenoid[illegible] dioxygenase
CYP707A3	AT5G45340	CLAR_DN44841_c0_g1	ctcagtaggaaatgtgggtcttg	gcataacttcatccctcggaag	Cytochrome P4[illegible] family 707
CYP707A3	AT5G45340	CLAR_DN43170_c0_g2	ccccagataagtcaaaacatcg	tggctatcaacgtcgcatc	Cytochrome P4[illegible] family 707
CYP707A3	AT5G45340	CLAR_DN43349_c0_g1	ttgtctcttacgctgtgtcatcg	tgtgggaagaaggcggtttatg	Cytochrome P4[illegible] family 707
CYP707A3	AT5G45340	CLAR_DN44491_c1_g1	gatgcctccaaagcctaacag	gtggtggatcaggacgagtatc	Cytochrome P4[illegible] family 707
AAO3	AT2G27150	CLAR_DN54372_c1_g1	aagtgtggctgttgggtttc	tggtttacagggccatttctc	aldehyde oxidas[illegible]
ABA1	AT5G67030	CLAR_DN50982_c0_g1_B	ggctgaacgaagcgacatactc	cactcccagcaggttcattgtg	zeaxanthin epoxidase (ZEP[illegible]
LisH dimerisation motif	AT4G32551	CLAR_DN46563_c1_g1	ggaagttgggacgataaaagc	cggtaatcaaatggttgtgc	**HOUSEKEEPI[illegible]**

NCED	AT3G14440	TBAR_DN117190_c1_g5	gcctctcttctcgctatgtcc	caaaccctcgtgccgtctatc	9-cis-epoxycarotenoid dioxygenase
NCED	AT3G14440	TBAR_DN105025_c1_g1	tagttgacggaggatggagtg	cgacagaggacgctgcttttc	9-cis-epoxycarotenoid dioxygenase
CYP707A3	AT5G45340	TBAR_DN122486_c1_g3_B	cagctcaagataccaccgcc	gttctgtcgtgacagcctgg	Cytochrome P4[illegible] family 707
CYP707A3	AT5G45340	TBAR_DN113007_c0_g2	aggtgtcaaatggtgcttgg	atcccctccctcgtctctg	Cytochrome P4[illegible] family 707
AAO3	AT2G27150	TBAR_DN123260_c4_g3	agtctacagctcaagccagaacc	gacgcttcagcttgtatgcag	aldehyde oxidas[illegible]
ABA1	AT5G67030	TBAR_DN109993_c1_g1	tcgtcaatgcctggttcaatc	ccttccgaccaacttgtactg	zeaxanthin epoxidase (ZEP[illegible]
LisH dimerisation motif	AT4G32551	TBAR_DN113162_c2_g5_B	tccgctactcttcagcaggttc	gcctctgaggtaccagatcgac	**HOUSEKEEPI[illegible]**

Supplementary table 4. Study species and essential information. **Age**: years in seedbed + years after transplant to open field or pot. **Provenance**: Country of origin of the material. **Type of material**: trees from Seed Orchard progeny, Germplasm, field stand or planting stock. **Provider**: company providing the material.

**Species name**	**Size (cm)**	**Age (years)**	**Provenance**	**Type of material**	**Provider**
Picea sitchensis	20-50	2+1	Denmark	Orchard	Maelor
P. sitchensis x P. glauca	20-50	1+1	UK	Orchard	Maelor
Larix eurolepis	20-40	1+1	Denmark	Seed	Maelor
Tsuga heterophylla	30-40	1U1	USA	Seed	Maelor
Pseudotsuga menziesii	20-40	1+1	France	Orchard	Maelor
Chamaecyparis lawsoniana	20-40	1.5	UK	Stand	Alba
Juniper communis	20-40	1.5	UK	Planting stock	Alba
Taxus baccata	20-40	2	UK	Planting stock	Alba

Supplementary table 5. Summary of the linear model of **MWP** with **SWC**. **Blocks** were considered as random factor. Fixed part of the model includes: **Estimate** = Slope estimate, **CI** = Confidence interval, **p** = p-value.

z MWP

Predictors	Estimates	CI	p
(Intercept)	-17.57	-20.80 – -14.34	<0.001
Species [L. eurolepis]	1.11	-4.34 – 6.56	0.688
Species [P. sitchensis]	-9.62	-14.42 – -4.82	<0.001
Species [P. sitchensis x P. glauca]	-11.21	-15.67 – -6.75	<0.001
Species [S. sempervirens]	-10.27	-14.38 – -6.16	<0.001
Species [P. menziesii]	-13.69	-18.55 – -8.83	<0.001
Species [T. baccata]	-13.66	-18.12 – -9.20	<0.001
Species [J. communis]	-11.97	-16.69 – -7.26	<0.001
Species [C. lawsoniana]	-20.86	-25.35 – -16.36	<0.001
SWC	0.17	0.02 – 0.32	0.025
Species [L. eurolepis] * SWC	0.06	-0.24 – 0.35	0.699
Species [P. sitchensis] * SWC	0.36	0.13 – 0.59	0.002
Species [P. sitchensis x P. glauca] * SWC	0.40	0.17 – 0.63	0.001
Species [S. sempervirens] * SWC	0.54	0.29 – 0.80	<0.001
Species [P. menziesii] * SWC	0.60	0.33 – 0.88	<0.001
Species [T. baccata] * SWC	0.65	0.41 – 0.88	<0.001
Species [J. communis] * SWC	0.70	0.42 – 0.97	<0.001
Species [C. lawsoniana] * SWC	1.42	1.10 – 1.73	<0.001
Observations	239		
R^2 / R^2 adjusted	0.720 / 0.699		

Figures

“NCED_alignment_supplemental.pdf”

Supplementary figure 1. MAFFT multiple sequence alignment (**MSA**) of putative and model **NCED** protein sequences. **Amtr**: *A. trichopoda*; **At**: *A. thaliana*; **Cl**: *C. lawsoniana*; **Gb**: *G. biloba*; **Ll**: *L. laricina*; **Mp**: *M. polymorpha*; **Os**: *O. sativa*; **Pg**: *P. glauca*; **Ps**: *P. sitchensis*; **Sm**: *S. moellendorffii*; **To**: *T. occidentalis*. Residues with similarity scores above the 0.7 similarity cut-off value are shown in red on white background. Columns that are strictly conserved are shown in white on red background.

Supplementary figure 2. Putative **ABA1** orthologs ML phylogenetic tree. Support bootstrap values (1000 replicates) are shown on the nodes. **Red**: angiosperms; **Green**: gymnosperms; **Yellow**: seedless plants. **Amtr**: *A. trichopoda*; **At**: *A. thaliana*; **Cl**: *C. lawsoniana*; **Gb**: *G. biloba*; **Ll**: *L. laricina*; **Mp**: *M. polymorpha*; **Os**: *O. sativa*; **Pg**: *P. glauca*; **Ps**: *P. sitchensis*; **Sm**: *S. moellendorffii*; **To**: *T. occidentalis*.

"ABA1_alignment_supplemental.pdf"

Supplementary figure 3. MAFFT multiple sequence alignment (**MSA**) of putative and model **ABA1** protein sequences. **Amtr**: *A. trichopoda*; **At**: *A. thaliana*; **Cl**: *C. lawsoniana*; **Gb**: *G. biloba*; **Ll**: *L. laricina*; **Mp**: *M. polymorpha*; **Os**: *O. sativa*; **Pg**: *P. glauca*; **Ps**: *P. sitchensis*; **Sm**: *S. moellendorffii*; **To**: *T. occidentalis*. Residues with similarity scores above the 0.7 similarity cut-off value are shown in red on white background. Columns that are strictly conserved are shown in white on red background.

Supplementary figure 4. Putative AAO3 orthologs ML phylogenetic tree. Support bootstrap values (1000 replicates) are shown on the nodes. **Red**: angiosperms; **Green**: gymnosperms; **Yellow**: seedless plants. **Amtr**: *A. trichopoda;* **At**: *A. thaliana;* **Cl**: *C. lawsoniana;* **Gb**: *G. biloba;* ***Ll***: *L. laricina;* **Mp**: *M. polymorpha;* **Os**: *O. sativa;* **Pg**: *P. glauca;* **Ps**: *P. sitchensis;* **Sm**: *S. moellendorffii;* **To**: *T. occidentalis.*

"AAO3_alignment_supplemental.pdf"

Supplementary figure 5. MAFFT multiple sequence alignment (**MSA**) of putative and model **AAO** protein sequences. **Amtr**: *A. trichopoda*; **At**: *A. thaliana*; **Cl**: *C. lawsoniana*; **Gb**: *G. biloba*; **Ll**: *L. laricina*; **Mp**: *M. polymorpha*; **Os**: *O. sativa*; **Pg**: *P. glauca*; **Ps**: *P. sitchensis*; **Sm**: *S. moellendorffii*; **To**: *T. occidentalis*. Residues with similarity scores above the 0.7 similarity cut-off value are shown in red on white background. Columns that are strictly conserved are shown in white on red background.

Supplementary figure 6. Putative **CYP707A** orthologs ML phylogenetic tree. Support bootstrap values (1000 replicates) are shown on the nodes. **Red**: angiosperms; **Green**: gymnosperms; **Yellow**: seedless plants. **Amtr**: *A. trichopoda*; **At**: *A. thaliana*; **Cl**: *C. lawsoniana*; **Gb**: *G. biloba*; **Ll**: *L.*

laricina; **Mp**: *M. polymorpha*; **Os**: *O. sativa*; **Pg**: *P. glauca*; **Ps**: *P. sitchensis*; **Sm**: *S. moellendorffii*; **To**: *T. occidentalis*.

“CYP707A_alignment_supplemental.pdf”

Supplementary figure 7 MAFFT multiple sequence alignment (**MSA**) of putative and model **CYP707A** protein sequences. **Amtr**: *A. trichopoda*; **At**: *A. thaliana*; **Cl**: *C. lawsoniana*; **Gb**: *G. biloba*; **Ll**: *L. laricina*; **Mp**: *M. polymorpha*; **Os**: *O. sativa*; **Pg**: *P. glauca*; **Ps**: *P. sitchensis*; **Sm**: *S. moellendorffii*; **To**: *T. occidentalis*. Residues with similarity scores above the 0.7 similarity cut-off value are shown in red on white background. Columns that are strictly conserved are shown in white on red background.

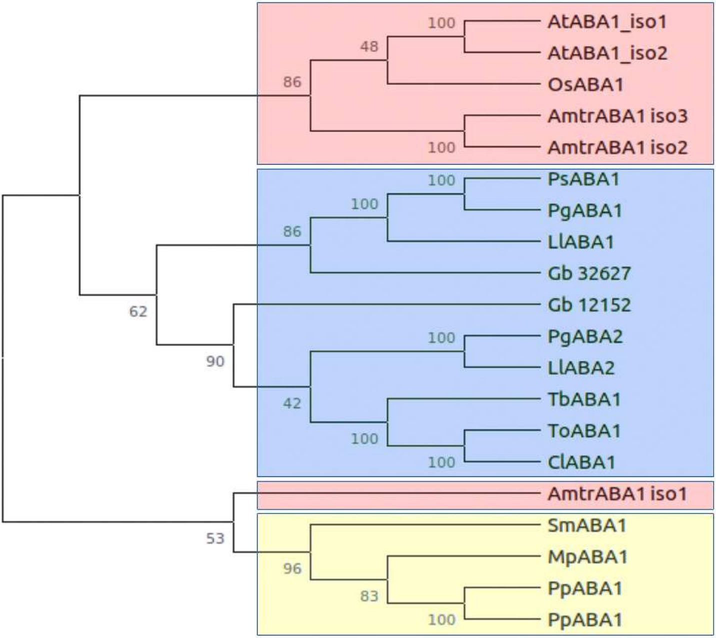

AtABA1_iso1
AtABA1_iso2
OsABA1
AmtrABA1 iso3
AmtrABA1 iso2
PsABA1
PgABA1
LlABA1
Gb 32627
Gb 12152
PgABA2
LlABA2
TbABA1
ToABA1
ClABA1
AmtrABA1 iso1
SmABA1
MpABA1
PpABA1
PpABA1
100
48
86
100
100
100
86
62
90
100
42
100
100
53
96
83
100

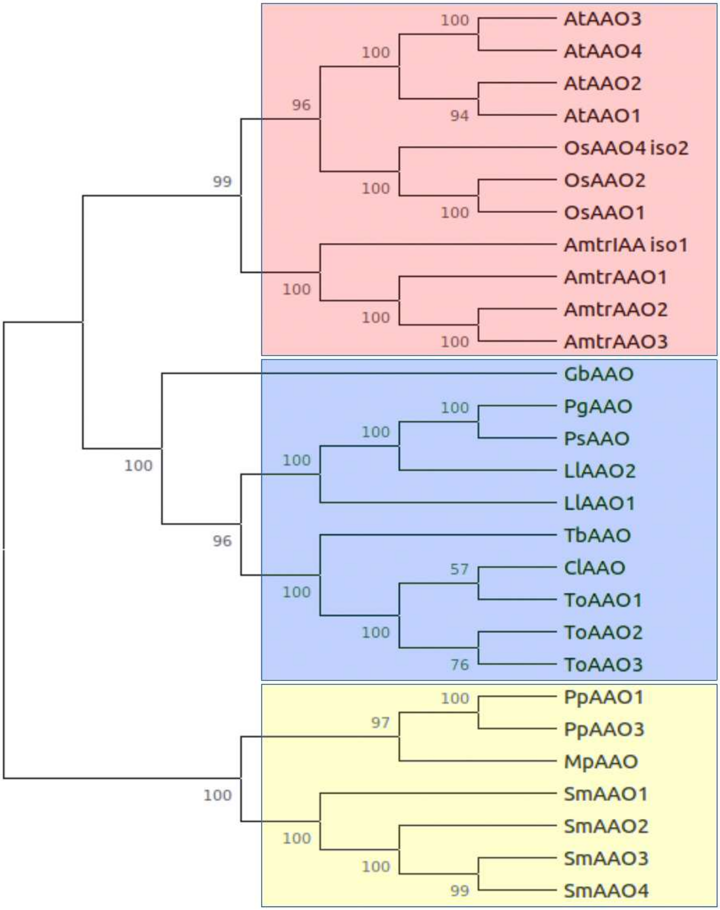

AtAAO3
AtAAO4
AtAAO2
AtAAO1
OsAAO4 iso2
OsAAO2
OsAAO1
AmtrIAA iso1
AmtrAAO1
AmtrAAO2
AmtrAAO3
GbAAO
PgAAO
PsAAO
LlAAO2
LlAAO1
TbAAO
ClAAO
ToAAO1
ToAAO2
ToAAO3
PpAAO1
PpAAO3
MpAAO
SmAAO1
SmAAO2
SmAAO3
SmAAO4
100
100
96
94
99
100
100
100
100
100
100
100
100
100
100
96
57
100
100
76
100
97
100
100
100
99

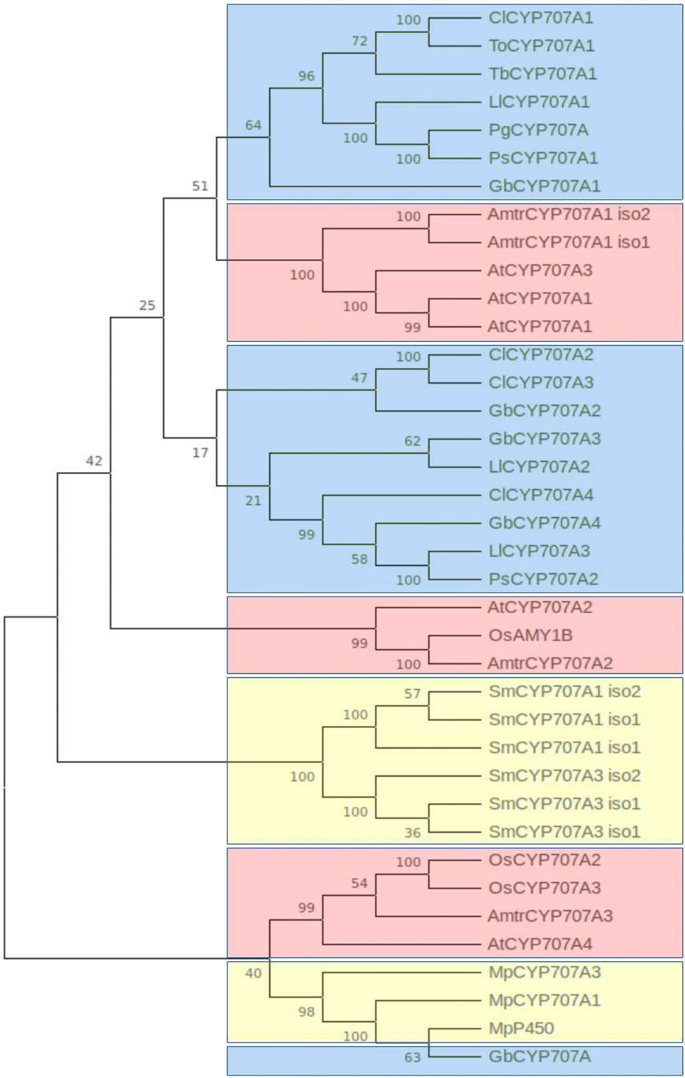

ClCYP707A1
ToCYP707A1
TbCYP707A1
LlCYP707A1
PgCYP707A
PsCYP707A1
GbCYP707A1
AmtrCYP707A1 iso2
AmtrCYP707A1 iso1
AtCYP707A3
AtCYP707A1
AtCYP707A1
ClCYP707A2
ClCYP707A3
GbCYP707A2
GbCYP707A3
LlCYP707A2
ClCYP707A4
GbCYP707A4
LlCYP707A3
PsCYP707A2
AtCYP707A2
OsAMY1B
AmtrCYP707A2
SmCYP707A1 iso2
SmCYP707A1 iso1
SmCYP707A1 iso1
SmCYP707A3 iso2
SmCYP707A3 iso1
SmCYP707A3 iso1
OsCYP707A2
OsCYP707A3
AmtrCYP707A3
AtCYP707A4
MpCYP707A3
MpCYP707A1
MpP450
GbCYP707A
